# Tandem mechanochemical engineering yields highly crystalline metal–organic frameworks

**DOI:** 10.1039/d5sc07662e

**Published:** 2025-12-29

**Authors:** Zhuorigebatu Tegudeer, Wen-Yang Gao

**Affiliations:** a Department of Chemistry and Biochemistry, Nanoscale & Quantum Phenomena Institute, Ohio University Athens Ohio 45701 USA gaow@ohio.edu

## Abstract

The formation of highly crystalline metal–organic frameworks (MOFs) relies on reversible metal–ligand (M–L) bond formation under conditions that enable defect annealing. While solvothermal synthesis remains the most common method for producing crystalline MOFs, mechanochemical synthesis is emerging as a greener alternative. However, the solid-state nature of mechanochemical reactions—even when assisted by catalytic amounts of liquid additives—limits molecular mobility, thereby impeding defect annealing and crystallization. This work introduces a tandem mechanochemical engineering strategy to achieve highly crystalline MOFs by incorporating a second class of reversible bond formation—imine condensation—alongside traditional M–L coordination. The critical role of cooperative dynamics between M–L and imine reversible bonds is highlighted by systematic investigations using similar high-connectivity ligand analogues featuring irreversible covalent linkages (*e.g.*, ether, amide, or alkyne), which fail to produce quality crystalline MOF phases under mechanochemical conditions. The synergistic effect of dual reversible bonds addresses sluggish reaction kinetics inherent to solid-state processes, enhances crystallization kinetics, and enables the efficient mechanochemical synthesis of MOFs with improved crystallinity under ambient conditions, particularly for frameworks constructed from high-connectivity ligands.

## Abstract 2

Tandem mechanochemical engineering, combining reversible M–L coordination and imine condensation, overcomes sluggish reaction kinetics and enables direct one-pot synthesis of highly crystalline MOFs in seconds.

## Graphical abstract

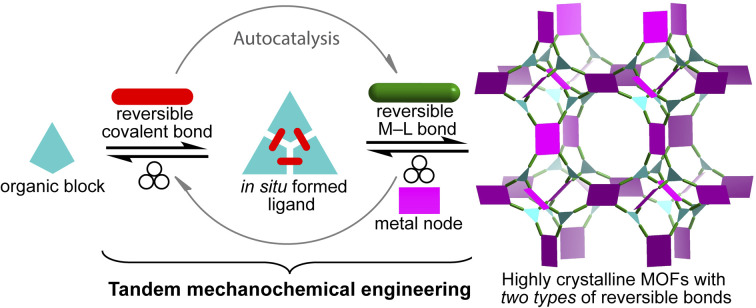

## Introduction

**Fig. 1:**
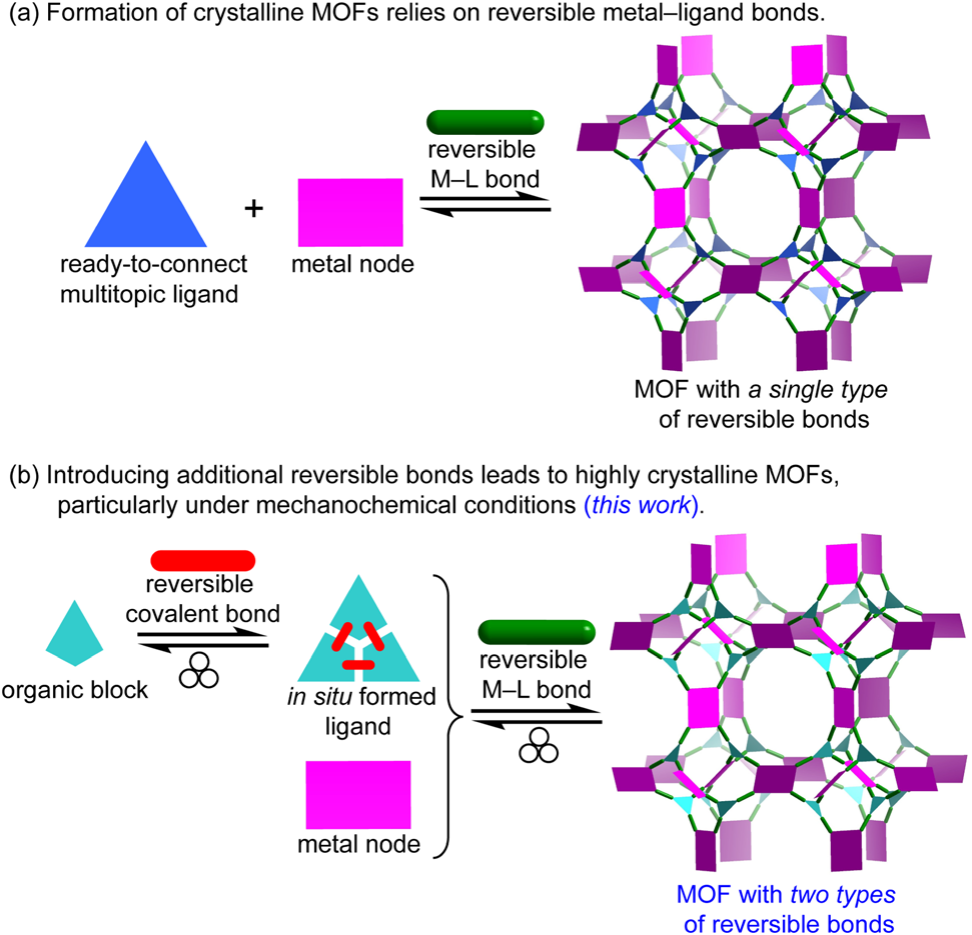
(a) Conventional MOF synthesis typically relies on a single type of reversible dative bonds between metal ions and organic ligands. This reversibility enables defect annealing and facilitates the formation of crystalline MOFs. (b) This work introduces a synthetic strategy that incorporates reversible covalent bonds alongside the M–L dative bonds during mechanochemical MOF synthesis. The synergy between these two types of reversible interactions is expected to enhance the crystallinity of MOFs prepared *via* mechanochemistry.

The formation of highly crystalline metal–organic frameworks (MOFs) hinges on reversible metal–ligand (M–L) dative bond formation between ready-to-connect organic ligands and metal ions under appropriate synthetic conditions (Fig. 1a).^1–4^ Traditionally, solvothermal methods dominate MOF synthesis, where reactants are dissolved in excess organic solvents and incubated at elevated temperatures over extended periods.^5–7^ These conditions allow the system to reach thermodynamic equilibrium, enabling defect annealing and promoting the growth of well-ordered crystalline phases.

As a more sustainable alternative, mechanochemical synthesis has emerged, using mechanical energy to drive MOF assembly under solvent-free conditions or in the presence of only catalytic amounts of solvents.^8–19^ However, the (near) solid-state nature of mechanochemistry limits molecular mobility and typically reduces the reversibility of M–L bonds. Our previous studies have shown that ligand exchange kinetics significantly impact the crystallinity of mechanochemically synthesized MOFs, and that relatively inert metals, coupled with low M–L bond reversibility, often yield only small crystallites.^20,21^ This constraint—the decreased reversibility of M–L bonds under solid-state conditions—poses a particular challenge for incorporating bulky or inherently sluggish ligands that require dynamic chemical environments to assemble into ordered MOF structures. This explains why multitopic ligands with 2 to 4 connection points have been mechanochemically integrated into MOFs,^22–40^ but ligands with higher connectivity are rarely encountered in mechanochemistry.^41^ Herein, we propose tandem mechanochemical engineering as a new synthetic strategy to overcome this challenge and enable the efficient incorporation of bulky or less mobile ligands (*e.g.*, high-connectivity multitopic ligands) into MOFs under mechanochemical conditions (Fig. 1b). Our earlier study showed that imines can be directly introduced into MOFs by mechanochemistry.^42^ In contrast, the present work departs from that approach to tackle the sluggish reaction kinetics inherent to solid-state processes during crystalline phase formation. By introducing a second type of reversible bonds, namely a dynamic covalent imine bond, alongside the M–L dative bond, this tandem approach enables the one-pot mechanochemical synthesis of highly crystalline MOFs from high-connectivity ligands. Remarkably, it also reduces reaction time by approximately 100-fold (*e.g.*, from typical 1.5 hours to 56 seconds or less). The use of multiple reversible interactions mitigates the limited molecular mobility inherent to solventless conditions and significantly expands the scope of mechanochemical synthesis to a broader family of MOFs with diverse topologies.

**Fig. 2:**
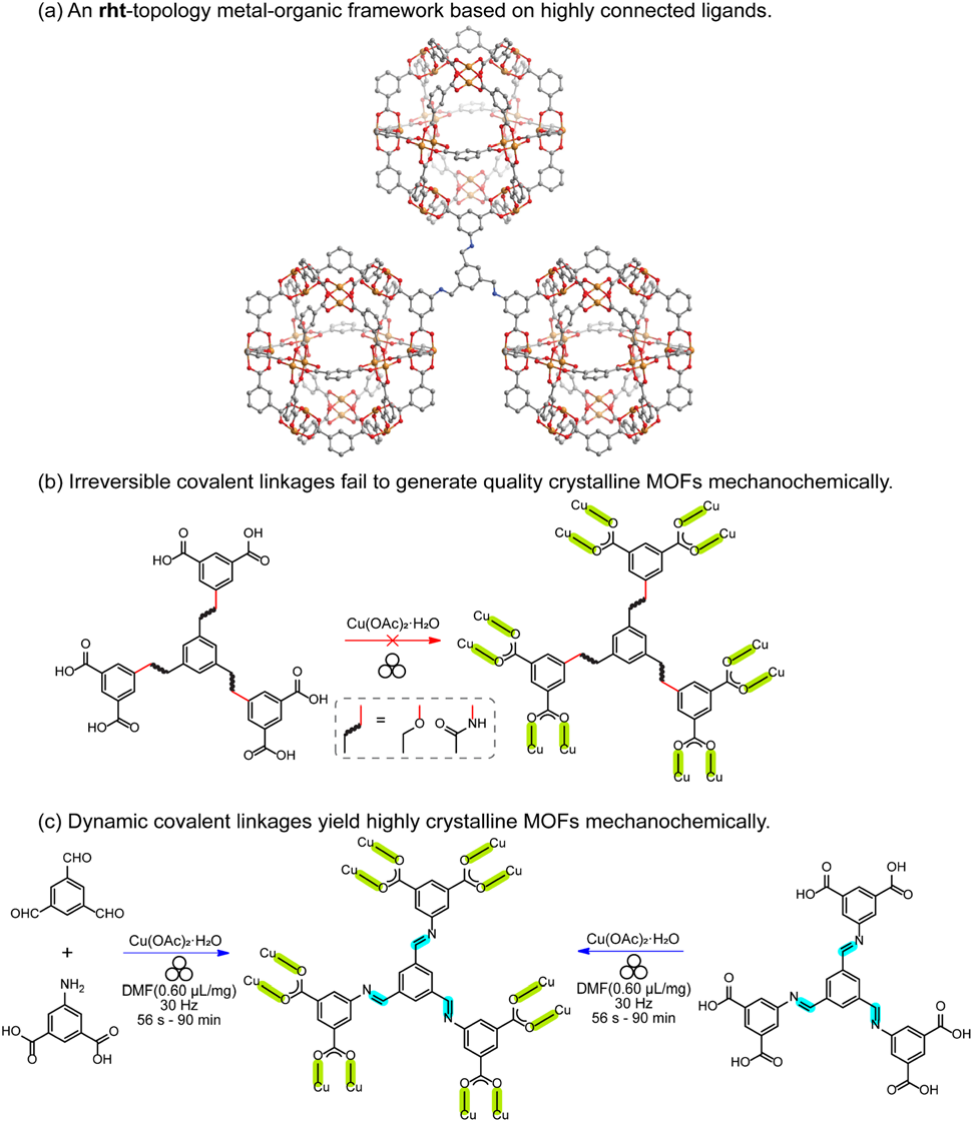
(a) An example of the rht-topology MOF is composed of hexacarboxylic ligands and metal paddlewheel dimers. This network can be simplified into 3-connected organic nodes and 24-connected cuboctahedra composed of 24 isophthalate moieties and 12 dinuclear copper paddlewheel units. (b) Two hexacarboxylic ligands featuring ether and amide linkages—both known to yield rht-topology MOFs *via* solvothermal synthesis—were extensively tested under various mechanochemical milling conditions, but failed to produce high-quality crystalline MOFs. (c) Incorporating reversible covalent linkages (*e.g.*, imine) into highly connected ligands mitigates the irreversibility of M–L interactions by effectively reducing ligand connectivity. This strategy enables the successful formation of a highly crystalline rht-topology MOF, either through a one-pot cascade reaction or by using a pre-synthesized imine-based hexacarboxylic ligand. All the reversible bonds, including Cu–O and imine linkages, are highlighted.

As a proof-of-concept, we selected rht-topology MOFs (Fig. 2a), which are typically constructed from highly connected hexacarboxylic ligands and metal paddlewheel dimers (*e.g.*, Cu_2_(OOC)_4_).^43–50^ The (3,24)-connected rht-topology network represents a canonical example of reticular chemistry, employing supermolecular building blocks—specifically, 24-connected cuboctahedra composed of 24 isophthalate moieties and 12 dinuclear copper paddlewheel units.^51^ These frameworks exemplify best practices in MOF reticular design, achieving record-high surface areas and free pore volumes through ligand expansion while avoiding framework interpenetration.^52–54^ However, to the best of our knowledge, no rht-topology MOFs has been synthesized mechanochemically. While the fast kinetics of Cu–O bond formation have enabled the mechanochemical assembly of copper paddlewheel-based MOFs^26,29,55,56^ (*e.g.*, HKUST-1,^57^ MOF-14,^58^ and MOF-505 (ref. 59)) using tricarboxylic or tetracarboxylic ligands, the incorporation of hexacarboxylic ligands remain a significant challenge under mechanochemical conditions. This difficulty is tentatively attributed to the high lattice energy barrier that must be overcome during the annealing of M–L defects involving metal nodes and high-connectivity ligands.

## Results and discussion

**Fig. 3:**
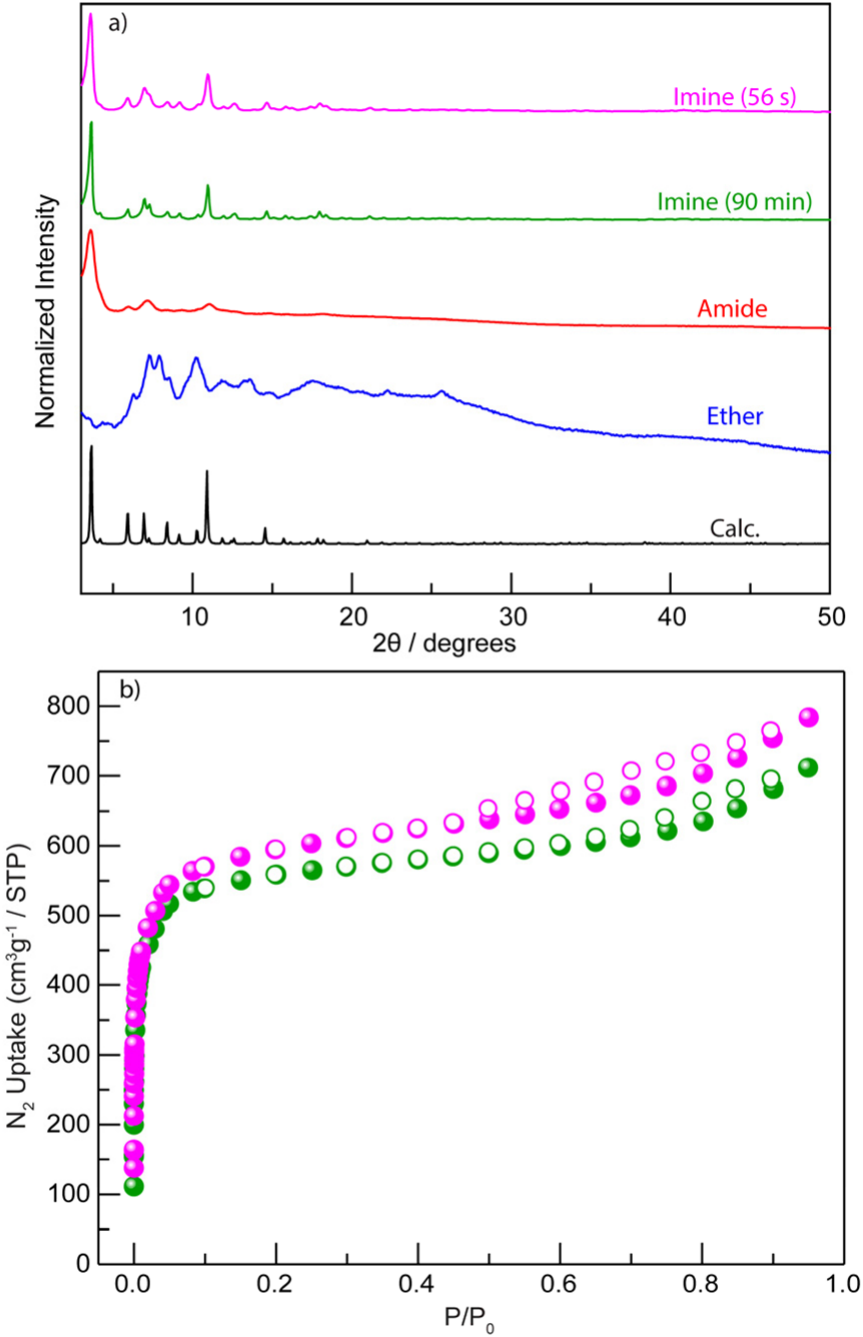
(a) PXRD patterns of mechanochemically obtained solids derived from ether- (blue), amide- (red), imine-linked ligands (90 min, green and 56 s, magenta) are compared to the calculated pattern for the amide-linked MOF (black). (b) N_2_ adsorption isotherms at 77 K were collected for imine-based rht-topology MOFs prepared mechanochemically (90 min, green dot; 56 s, magenta dot).

Two hexacarboxylic ligands featuring ether and amide linkages (Fig. 2b)—both previously known to yield rht-topology MOFs *via* solvothermal synthesis^47,60,61^—were extensively attempted for mechanochemical assembly under various milling conditions. However, powder X-ray diffraction (PXRD, Table S1 and Fig. 3a and S1) reveal poorly crystalline phases for the ether-linked ligand. The amide-linked ligand produced relatively broad PXRD patterns matching the calculated rht structure under certain conditions (Table S2 and Fig. 3a and S2). Nevertheless, N_2_ adsorption isotherms at 77 K (Fig. S3) showed significantly reduced Brunauer–Emmett–Teller (BET) surface areas compared to those obtained from solvothermal synthesis (215–1069 m^2^ g^−1^*vs.* 3160 m^2^ g^−1^).^61^ The parameter scope of our mechanochemical trials included variation in the type and amount of liquid additive (*e.g.*, *N*,*N*-dimethylformamide, DMF), milling time, and milling frequency. Moreover, we also tested high-temperature ball-milling conditions^62^—blowing 80 °C and 120 °C hot air onto the stainless-steel milling jars–for reactions involving both the amide- and ether-linked ligands, in order to evaluate how elevated temperature influences reagent mobility in the solid state and the resulting crystallinity during the MOF assembly. However, no improvement in the PXRD patterns (Fig. S4) was observed, and reflections corresponding to elemental Cu appeared instead. These unsuccessful attempts underscore the challenge of incorporating high-connectivity ligands into crystalline MOFs under mechanochemical conditions, likely due to the reduced reversibility of M–L bond formation in the (near) solid environment and other potential deleterious reactions observed for Cu(ii).

In contrast to the previously tested irreversible covalent linkages (*e.g.*, ether and amide), we propose that incorporating reversible or dynamic covalent linkage (*e.g.*, imine) into highly connected ligands can mitigate the irreversibility of M–L interactions by effectively reducing the number of ligand connectivity during the assembly process (Fig. 2c). This enhanced reversibility is expected to facilitate the mechanochemical crystallization of MOFs from otherwise sluggish, highly connected ligands. Therefore, we initiated the mechanochemical synthesis of an rht-topology MOF by milling trimesaldehyde, 5-aminoisophthalic acid, and Cu(OAc)_2_·H_2_O in a molar ratio of 1 : 3 : 3 in the presence of DMF (*η* = 0.60 µL mg^−1^, Table S3 and Fig. S5) using a stainless-steel milling cup and two stainless steel balls. The reaction mixture was milled at 30 Hz for 90 min. The resultant blue solids were washed with acetone and collected by centrifugation. PXRD analysis (Fig. 3a) confirmed the phase purity of the obtained imine-derived rht-topolgy MOF, based on the excellent agreement between the experimental patterns and the simulated ones (calculated from the amide-linked framework). Additionally, we synthesized the imine-based hexacarboxylic ligand under standard solution-phase condensation conditions catalyzed by acetic acid (see details in SI) and used it for the mechanochemical synthesis of the above rht-MOF. The isolated imine ligand was confirmed by ^1^H NMR (Fig. S6), high-resolution mass spectrometry (HRMS), and infrared spectroscopy (Fig. S7). The mechanochemical MOF product obtained from this pre-synthesized imine ligand was identified by PXRD (Fig. S8) as the same phase as the rht-MOF prepared *via* the one-pot cascade reaction. This observation is consistent with previous findings that mechanochemical synthesis can deliver crystalline imine-based covalent organic frameworks^63–67^ and MOFs,^42^ and further demonstrates the imine formation and cleavage remain reversible under the applied mechanochemical conditions. In contrast, similar mechanochemical reactions employing the hexacarboxylic ligands with irreversible covalent ether or amide linkages failed to yield the targeted high-quality crystalline MOFs. This comparison underscores the effectiveness of our tandem mechanochemical engineering strategy, where the introduction of an additional class of reversible bonds significantly improves the crystallization of MOFs under mechanochemical conditions.

Moreover, due to the hydrolytic instability of the imine motif,^68,69^ imine-based complexes and MOFs are well-suited for direct mechanochemical synthesis from aldehyde and primary amine precursors, along with the metal source.^42,70–72^ We attempted a series of solvothermal reactions using the pre-synthesized imine-based hexacarboxylic acid ligand (see details in SI, Table S4, and Fig. S9) to access this rht-MOF. However, the yields were very low, or the reactions were unsuccessful. Consistent with the lability of the imine linkages, HRMS analysis of reaction solutions indicated that the ligand decomposed back into its aldehyde and amine precursors, particularly in water-rich environment. In contrast, the tandem mechanochemical approach not only avoids the decomposition pathways that often occur under solvothermal conditions, but also enables the formation of the first imine-based rht-MOF among its various analogues, eliminating the need for the tedious pre-synthesis of imine ligands.

Additional characterizations of the imine-based rht-MOF obtained by tandem mechanochemical synthesis include IR spectroscopy, thermogravimetric analysis (TGA), and N_2_ adsorption analysis at 77 K. The IR spectrum (Fig. S10) shows that a strong peak emerged at 1371 cm^−1^, attributed to the coordinated C=O stretch, validating the formation of the desired MOF. The TGA analysis (Fig. S11) reveals that the obtained imine-based rht-topology MOF exhibits thermal stability comparable to other known rht-MOFs,^60,61^ which is stable up to 280 °C or above. Thus, the imine-based rht-MOF was activated by heating at 60 °C under high vacuum for 18 h prior to gas adsorption measurements. The permanent porosity of the imine-based rht-MOF was characterized by N_2_ adsorption isotherms at 77 K (Fig. 3b), which provided a BET surface area of 2238 m^2^ g^−1^ (*P*/*P*_0_ = 0.007–0.03). This value is somewhat lower than that of its amide analogue (3160 m^2^ g^−1^), likely due to the entrapment of molecular fragments from incomplete reactions within the cavities, which partially occupy the pore volume. Nevertheless, the BET surface area of the mechanochemically synthesized imine-based rht-MOF remains higher than that of the solvothermally obtained samples (Fig. S12), which exhibit BET surface area values ranging from 1244 to 1265 m^2^ g^−1^ under the same activation conditions.

Furthermore, to evaluate how the second type of reversible bond influences reaction time, we systematically examined the minimum milling duration required to synthesize the imine-based rht-MOF (Table S5). Whereas many mechanochemical studies employ ∼90 minutes milling times, well-defined PXRD patterns were observed in as little as 56 seconds (Fig. 3a), with all precursor-related PXRD peaks disappearing (Fig. S13). Remarkably, even after only 28 seconds, the MOF phase already dominates, with no apparent signatures of precursors. This rapid reaction yields materials of comparable quality to those produced at 90 min, as confirmed by N_2_ adsorption isotherms at 77 K (Fig. 3b). The BET surface area calculated from the 56 seconds product is 2278 m^2^ g^−1^ (*P*/*P*_0_ = 0.007–0.03), even slightly higher than that of the 90 min sample. This remarkably short reaction time highlights the critical role of dynamic imine bond in facilitating rapid MOF crystallization under mechanochemical conditions.

**Fig. 4:**
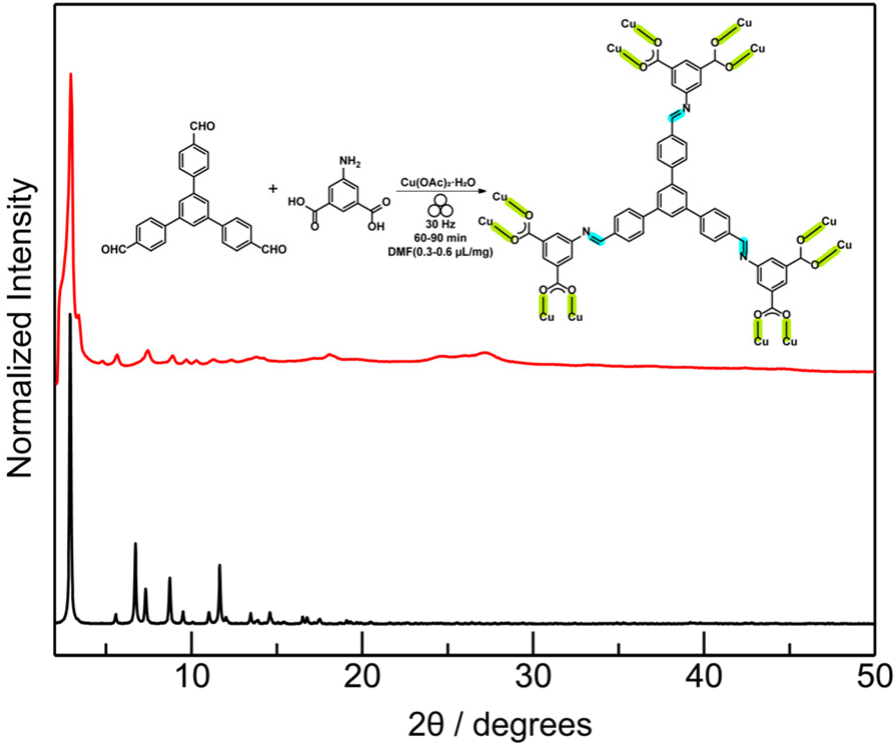
An expanded rht-topology MOF featuring the imine-linkage allows for mechanochemical synthesis, confirmed by PXRD patterns (red line, as-synthesized; black line, calculated from the amide-linked isostructural MOF).

Tandem mechanochemical engineering also enables us to synthesize an expanded analogue of the imine-based rht-MOF by replacing trimesaldehyde with 1,3,5-tris(4-formylphenyl)benzene under similar milling conditions. The obtained dark green-colored crystalline solids were explored (Table S6; Fig. S14) and confirmed by PXRD (Fig. 4), consistent with the pattern calculated from an amide-linked MOF analogue.^73^ The reaction progress was monitored by IR spectroscopy (Fig. S15), illustrating the appearance of coordinated carbonyl stretch at 1372 cm^−1^. TGA (Fig. S16) and N_2_ adsorption measurements (Fig. S17) provide additional characterization data on the mechanochemically obtained expanded rht-topology MOF. N_2_ adsorption isotherms at 77 K, which provided a calculated BET surface area of 976 m^2^ g^−1^ (*P*/*P*_0_ = 0.007–0.03). The decreased surface area value of the mechanochemically obtained large-pore MOF compared to that of the calculated one remains common,^21,29,55^ tentatively due to molecular fragments trapped in the expanded cavities. Control experiments to synthesize this expanded imine-linked rht-MOF under mechanochemical and solvothermal using the preformed imine-based expanded hexacarboxylic ligand were also attempted (Fig. S18–S21 and Table S7), which are consistent with observations in the last study.

**Fig. 5:**
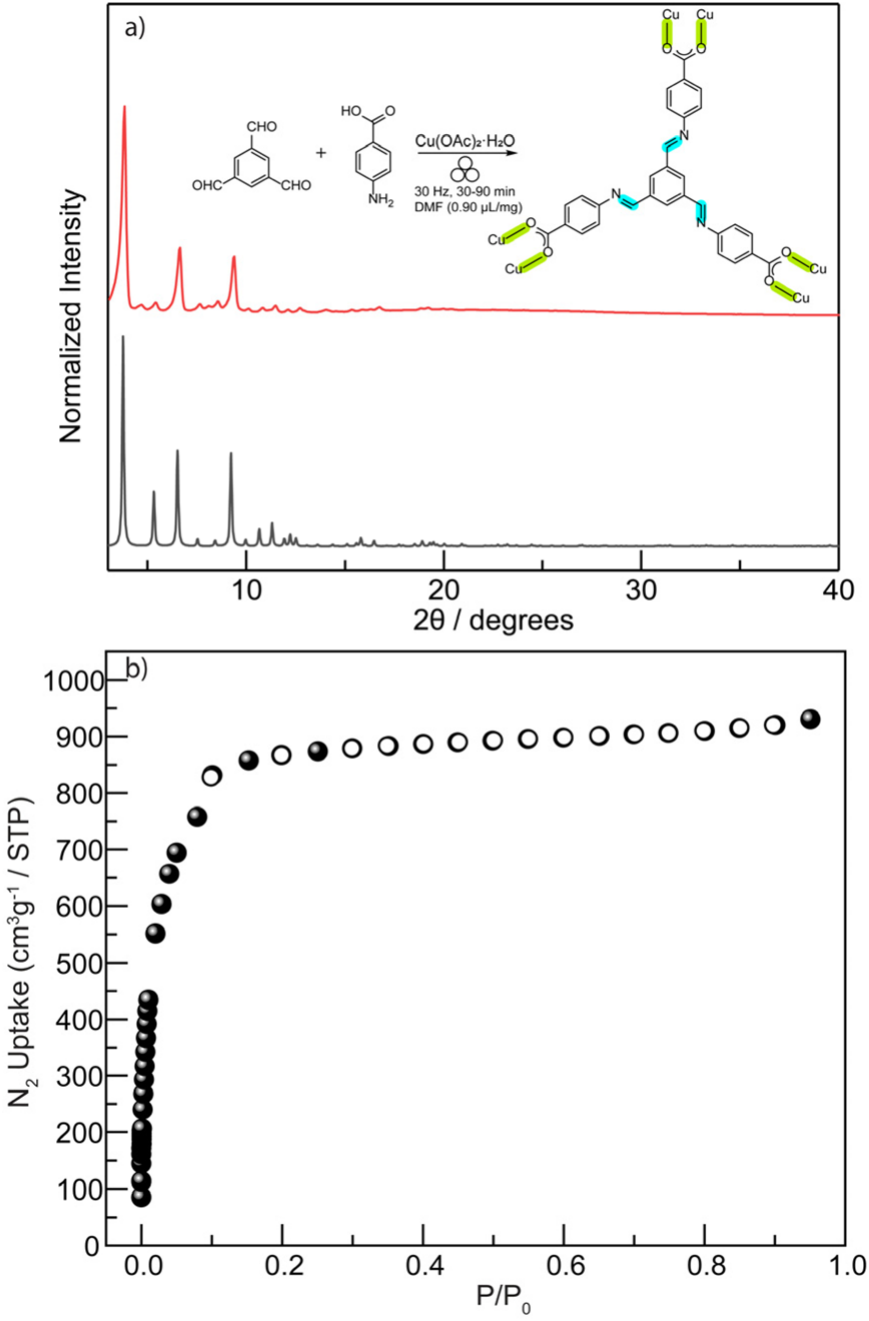
(a) The PXRD pattern of mechanochemically obtained solids derived from the imine-linked ligand (red line) is compared to the calculated pattern from the amide-linked pto-topolgy MOF (black line). (b) N_2_ adsorption isotherms at 77 K were collected for the imine-based pto-topolgy MOF prepared mechanochemically.

In addition to the rht-topology MOFs, we also applied tandem mechanochemical engineering to an imine-based pto-topology MOF (Fig. 5a), isostructural to MOF-14.^58^ Its direct mechanochemical synthesis was achieved by milling trimesaldehyde, 4-aminobenzoic acid, and Cu(OAc)_2_·H_2_O in a molar ratio of 2 : 6 : 3 with the addition of DMF (*η* = 0.90 µL mg^−1^, Fig. S22) for 60 min (Fig. S23 and Table S8). The harvested blue solids were characterized by a suite of solid-state characterizations, including PXRD, IR, TGA, and N_2_ adsorption analysis. The PXRD pattern (Fig. 5a) matches well with that of an amide-based pto-MOF indicating an isostructural lattice. The IR spectrum (Fig. S24) confirms the coordinated C=O stretch observed at 1400 cm^−1^ in the imine-linked pto-MOF illustrates the formation of dative bonds. In addition, the disappearance of N–H stretches between 3473 cm^−1^ and 3364 cm^−1^ from the primary amine precursor highlights its conversion to imine bonds. The TGA plot (Fig. S25) shows its thermal stability until 280 °C, comparable to the previous imine-based rht-MOF. Notably, while this imine-based pto-MOF can be synthesized mechanochemically using the preformed imine-based tricarboxylic ligand (Fig. S26–28), direct solvothermal synthesis fails (see details in SI and Table S9), largely due to the ready collapse of the imine ligand.

Though MOF-14 allows for its direct mechanochemical synthesis using 1,3,5-tris(4-carboxylphenyl)benzene (H_3_btb) and Cu(OAc)_2_·H_2_O,^29,55^ we have attempted a number of milling conditions using other extended tricarboxylic ligands featuring irreversible covalent linkages (*e.g.*, amide and alkyne) and not been able to generate any crystalline MOF phases (Tables S10–S11 and Fig. S29–S32). This is another highlight that tandem mechanochemical engineering streamlines the incorporation of bulky or sluggish ligands into MOFs. In addition, the solvothermally obtained MOF-14 analogues with the amide^74,75^ and alkyne^76–79^ linkages readily collapse upon solvent removal (even activated by supercritical CO_2_) and thus were not characterized by N_2_ adsorption analysis in the literature. In contrast, our mechanochemically built imine-based pto-MOF still demonstrates its permanent high porosity based on N_2_ adsorption analysis at 77 K (Fig. 5b). The BET surface area was calculated to be 3136 m^2^ g^−1^ (*P*/*P*_0_ = 0.007–0.03), representing the highest value reported to date for mechanochemically prepared porous materials.

**Fig. 6:**
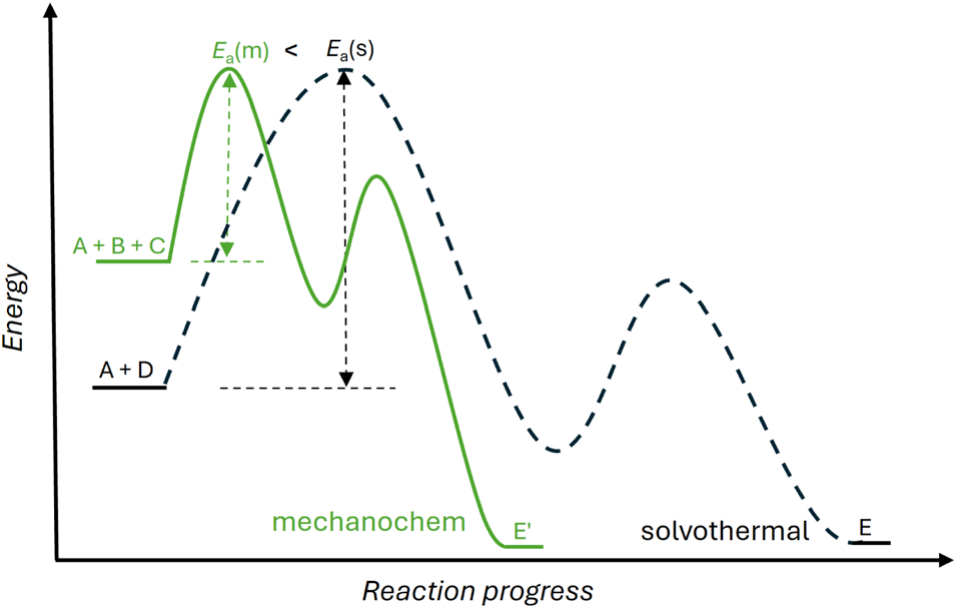
A schematic energy diagram comparing tandem mechanochemical engineering and solvothermal methods for crystal nucleation and growth steps. The tandem mechanochemical approach provides an alternative reaction pathway with reduced activation energies (green solid line), in contrast to the solvothermal method (black dash line) required for incorporating high-connectivity ligands.

The developed tandem mechanochemical engineering approach integrates two types of reversible bond formation processes into one single mechanochemical step, provides an alternative to bypass high lattice energy associated with multitopic ligands required to anneal defects, and incorporates hydrolytically unstable imine motifs into extended MOFs. The introduction of reversible dynamic covalent bonds coupled with the reversible M–L bond overcomes the sluggish reaction kinetics typically associated with solid-state reactions and expands the applicability of mechanochemical MOF synthesis. The byproduct of the reaction between carboxylic ligands and copper acetate is acetic acid, which is a known catalyst for imine condensation. Thus, the dative bond formation releases acetic acid, which accelerates the kinetics of imine bond formation and breakage. This process creates an autocatalytic cycle that promotes defect annealing and ultimately yields high-quality crystalline MOF phases. Fig. 6 presents a schematic energy diagram comparing the developed tandem mechanochemical engineering with traditional solvothermal methods. Incorporating high-connectivity ligands into crystalline MOF lattices typically requires high activation energy during crystal nucleation and growth, which is usually achievable only under solvothermal conditions. In contrast, introducing a second type of reversible bond in the mechanochemical synthesis segments the otherwise high activation barrier for bulky ligand assembly into a series of imine-mediated steps with lower individual barriers. This stepwise alternative pathway facilitates smoother kinetic transitions, accelerates crystallization, promotes defect annealing, and eventually drives the system toward the thermodynamically favored crystalline phases.

## Conclusions

In conclusion, we report a powerful synthetic strategy—tandem mechanochemical engineering—to access MOFs that are challenging under conventional mechanochemical conditions. The incorporation of a second type of reversible dynamic bonds (*e.g.*, imine) coupled with the M–L dative bonds yields highly crystalline MOFs in solid-state reactions, which also decreases reaction time to as little as 56 seconds. The synergy of M–L and imine reversible bonds promotes crystallization kinetics and facilitates efficient mechanochemical synthesis of MOFs with improved crystallinity under ambient conditions, especially those based on high-connectivity ligands. This strategy offers a general and efficient pathway for constructing well-ordered frameworks *via* solvent-free routes, while streamlining *in situ* ligand synthesis and MOF formation in a one-pot process.

## Author contributions

WG and ZT conceived and designed the project. ZT carried out the materials synthesis and characterization as well as performed data analysis. Both authors discussed the results and wrote the manuscript.

## Conflicts of interest

The authors declare the following competing financial interest(s): a provisional patent application has been filed based on the results reported in this manuscript. This patent application is held by Ohio University. The authors have no other relevant financial interests to disclose.

## Supplementary Material

SC-017-D5SC07662E-s001

## Data Availability

The data supporting this article have been included as part of the supplementary information (SI). Supplementary information: details of synthesis and characterization using powder X-ray diffraction, infrared spectroscopy, N_2_ adsorption isotherms, thermogravimetric analysis, and others. See DOI: https://doi.org/10.1039/d5sc07662e.
