# Supplementary material for: Tandem mechanochemical engineering yields highly crystalline metal–organic frameworks
Source: Chem Sci. 2025 Dec 29;17(8):4247–55. doi: 10.1039/d5sc07662e (PMC12787315; doi:10.1039/d5sc07662e)
Supplement: SC-017-D5SC07662E-s001 [file SC-017-D5SC07662E-s001.pdf]

Supplementary Information (SI) for Chemical Science.
This journal is © The Royal Society of Chemistry 2025

Supporting Information

Tandem Mechanochemical Engineering Enables the Formation of Highly Crystalline Metal-Organic Frameworks

Zhuorigebatu Tegudeer, and Wen-Yang Gao*

Department of Chemistry and Biochemistry, and Nanoscale & Quantum Phenomena Institute, Ohio University, Athens, Ohio 45701, United States. E-mail: gaow@ohio.edu

Table of Contents

A. General Considerations S3
B. Synthesis and Characterization S4
C. Supporting Data S22
D. References S60

A. General Considerations

Materials Solvents were obtained as ACS reagent grade and used without further purification. Unless otherwise noted, all chemicals and solvents were used as received. Copper(II) acetate monohydrate ($Cu(OAc)_2{\cdot}H_2O$), 4-aminobenzoic acid, 5-aminoisophthalic acid, benzene-1,3,5-tricarbaldehyde, 1,3,5-benzenetricarbonyl trichloride, 1,3,5-tris(4-carboxyphenyl)benzene (H_3btb), 1,3,5-tris(4-formylphenyl)benzene, and 4,4',4''-(benzene-1,3,5-triyltris(ethyne-2,1-diyl))tribenzoic acid were obtained from AmBeed. Acetic acid, acetone, and *N,N*-dimethylformamide (DMF) were purchased from Fisher Scientifc. Dimethyl solfuxide-d_6 (DMSO-d_6) was obtained from Cambridge Isotope Laboratories Inc. 200-proof ethanol was obtained from Decon Laboratories, Inc. Concentrated nitric acid and dichloromethane were purchased from VWR Chemicals. 5,5',5''-((Benzene-1,3,5-triyltris(methylene))tris(oxy))triisophthalic acid, 5,5',5''-((benzene-1,3,5-tricarbonyl)tris(azanediyl))triisophthalic acid, and 4,4',4''-((benzene-1,3,5-tricarbonyl)tris(azanediyl))tribenzoic acid were synthesized following reported procedures.[1, 2] UHP-grade N_2 and He, used in gas adsorption measurements, were obtained from Linde. All reactions were carried out under an ambient atmosphere unless otherwise noted.

Mechanochemical Synthesis Mechanochemical synthesis was conducted using a Retsch Mixer Mill MM 400. Starting materials were typically loaded into a 10-mL stainless-steel grinding jar with 2 stainless-steel grinding balls (10 mm Ø, 4.046±0.001 g) for the milling experiments. The yield of mechanochemical reactions was calculated solely from mass values using the fully activated (degas) method, as specified in *Gas Adsorption Details*, to exclude the impact of guest molecules.

Characterization Details NMR spectra were recorded on a Bruker Ultrashield 300 MHz. Spectra were referenced against residual proton solvent resonance: d_6-DMSO (2.50 ppm, ^{1}H).[3] ^{1}H NMR data are reported as follows: chemical shift (δ, ppm), (multiplicity: s(singlet), d(doublet), t(triplet), m(multiplet), br(broad)); coupling constant *J* in Hz; integration. Infrared (IR) spectra were recorded on a Thermo Scientific Nicolet iS20 DTGS. Spectra were blanked against KBr and determined by an average of 32 scans. IR data are reported as follows: wavenumber (cm^{-1}), (peak intensity: s, strong; m, medium; w, weak). Powder X-ray Diffraction (PXRD) measurements were carried out on a Rigaku Miniflex II (Cu Kα, 1.5406 Å; 40 kV, 15 mA). The angular range (2θ) was measured from 3.00 to 50.00° with a sampling width of 0.05° and a scan speed of 3.00° per minute. Simulated PXRD patterns were calculated using Mercury.[4] Thermogravimetric analysis (TGA) was performed on a Shimadzu DTG-60 analyzer with a ramping rate of 15 °C/min and a nitrogen flow rate of 50 mL/min.

Gas Adsorption Details N_2 adsorption isotherms (0−1.0 bar pressure range) were measured volumetrically at 77 K using an Anton Paar Autosorb-iQ. Each mechanochemically obtained solid sample was washed with acetone (15 mL × 3). Then the sample was transferred under N_2 atmosphere to a pre-weighed analysis tube. The sample of the amide-linked **rht**-MOF was evacuated at 120 °C until the outgas rate was <10 μbar/min and further maintained for 18 h. All other samples including the imine linkage-based MOFs and MOF-14 were evacuated at 60 °C until the outgas rate was <10 μbar/min and further maintained for 18 h. The analysis tube was weighed to determine the mass of the activated sample before the gas adsorption analysis. Brunauer-Emmett-Teller (BET) and Langmuir surface area values were calculated in the relative pressure range between 0.007 and 0.03.

Synthesis and Characterization

Mechanochemical attempts to synthesize the ether-linked rht-MOF

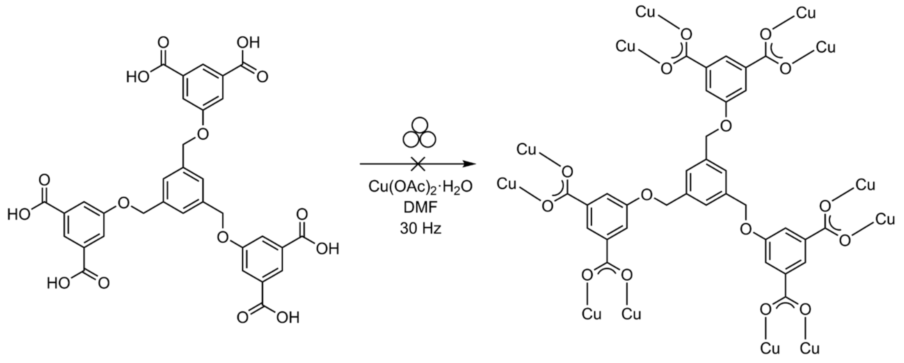

A 10-mL stainless steel grinding jar was charged with two stainless-steel grinding balls, 5,5',5''-((benzene-1,3,5-triyltris(methylene))tris(oxy))triisophthalic acid (0.062 g, 0.093 mmol, 1.0 equiv.), $Cu(OAc)_2{\cdot}H_2O$ (0.056 g, 0.280 mmol, 3.0 equiv.), and DMF (η = 0.60 or 0.90 µL/mg). The resulting mixture was milled at 30 Hz for various durations (Table S1). The solids obtained were collected and washed with acetone (15 mL × 3) before PXRD analysis (Figure S1). None of these typical milling conditions yielded the desired phase.

Table S1. Experimental parameters, including milling time and the amount of DMF additive, were varied in attempts to synthesize the ether-linked **rht**-MOF. The reaction conditions are summarized below, and corresponding PXRD patterns are shown in Figure S1.

Entry	Time / min	DMF additive (η / µL/mg, volume / µL)
1	60	0.60, 71
2	90	0.60, 71
3	90	0.90, 106
4	120	0.90, 106

Mechanochemical synthesis of the amide-linked rht-MOF

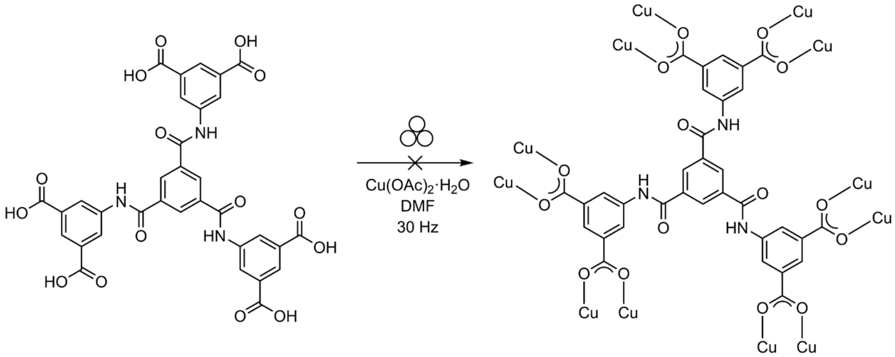

A 10-mL stainless steel grinding jar was charged with two stainless-steel grinding balls, 5,5',5''-((benzene-1,3,5-tricarbonyl)tris(azanediyl))triisophthalic acid (0.065 g, 0.093 mmol, 1.0 equiv.), $Cu(OAc)_2 \cdot H_2O$ (0.056 g, 0.280 mmol, 3.0 equiv.), and DMF (η = 0.60, 0.90 or 1.2 µL/mg). The resulting mixture was milled at 30 Hz for various durations (Table S2). The obtained solids were collected and washed with acetone (15 mL × 3) prior to PXRD analysis (Figure S2). N_2 adsorption isotherms at 77 K (Figure S3) were measured for samples showing well-defined PXRD patterns. However, the measured surface values (Table S13) remain much lower than those of the obtained solvothermally sample.

Table S2. Experimental parameters, including milling time and the amount of DMF additive, were varied in attempts to synthesize the amide-linked **rht**-MOF. The reaction conditions are summarized below, with corresponding PXRD patterns shown in Figure S2. (*) indicates samples selected for N_2 adsorption analysis.

Entry	Time / min	DMF additive (η / µL/mg, volume / µL)
1	30	0.60, 73
2*	60	0.60, 73
3	90	0.60, 73
4	120	0.60, 73
5	30	0.90, 109
6	60	0.90, 109
7*	90	0.90, 109
8*	120	0.90, 109
9	30	1.2, 145
10	60	1.2, 145
11	90	1.2, 145
12	120	1.2, 145

Elevated-temperature mechanochemical attempts to synthesize the ether-linked rht-MOF

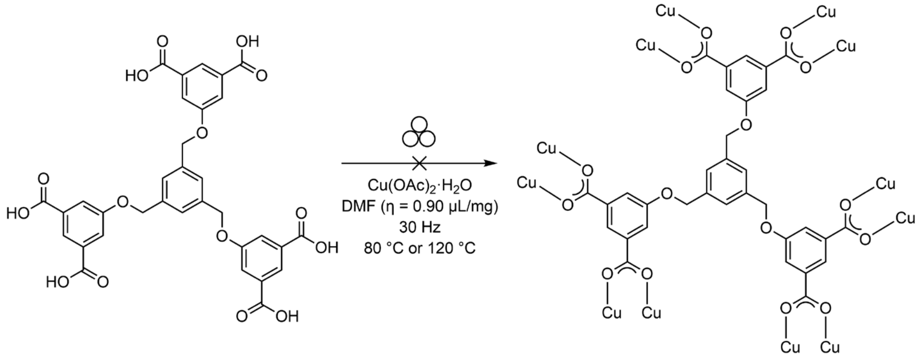

A 10-mL stainless steel grinding jar was charged with two stainless-steel grinding balls, 5,5',5''-((benzene-1,3,5-triyltris(methylene))tris(oxy))triisophthalic acid (0.062 g, 0.093 mmol, 1.0 equiv.), $Cu(OAc)_2 \cdot H_2O$ (0.056 g, 0.280 mmol, 3.0 equiv.), and DMF (η = 0.90 μL/mg, 106 μL). The resulting mixture was milled at 30 Hz for 120 min while externally heated with a heat gun to 80 °C or to 120 °C. The obtained solids were collected and washed with acetone (15 mL × 3) before PXRD analysis (Figure S4a). Neither of these conditions produced the desired phase, nor did they show improvement relative to the room-temperature trial.

Elevated-temperature mechanochemical synthesis of the amide-linked rht-MOF

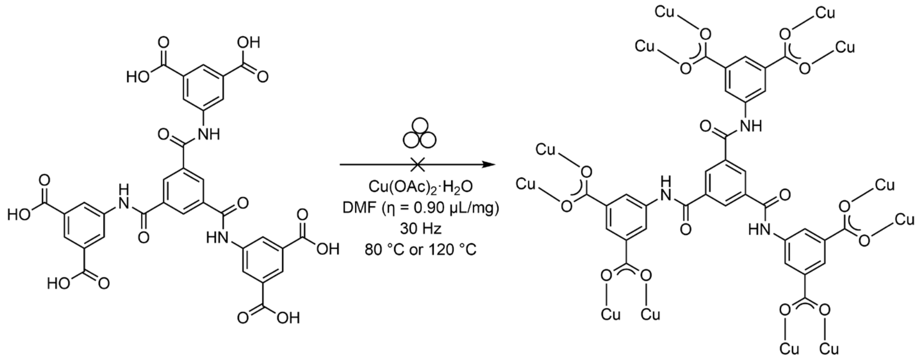

A 10-mL stainless steel grinding jar was charged with two stainless-steel grinding balls, 5,5',5''-((benzene-1,3,5-tricarbonyl)tris(azanediyl))triisophthalic acid (0.065 g, 0.093 mmol, 1.0 equiv.), $Cu(OAc)_2 \cdot H_2O$ (0.056 g, 0.28 mmol, 3.0 equiv.), and DMF (η = 0.90 μL/mg, 109 μL). The resulting mixture was milled at 30 Hz for 120 min while externally heated with a heat gun to 80 °C or to 120 °C. The obtained solids were collected and washed with acetone (15 mL × 3) prior to PXRD analysis (Figure S4b). Neither of these conditions produced the desired phase, nor did they show improvement relative to the room-temperature trial. Instead, reflections corresponding to elemental Cu (Figure S4c) were observed in the PXRD patterns.[5]

Screening of additive amounts for mechanochemical synthesis of the imine-linked rht-MOF

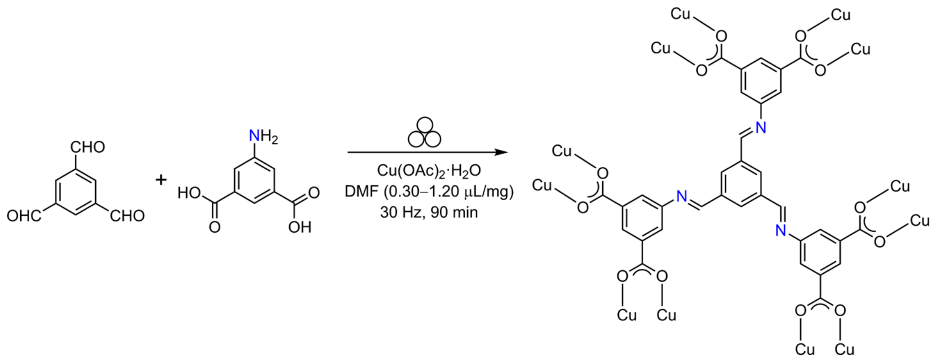

A 10-mL stainless steel grinding jar was charged with two stainless-steel grinding balls, benzene-1,3,5-tricarboxaldehyde (0.012 g, 0.074 mmol, 1.0 equiv.), 5-aminoisophthalic acid (0.040 g, 0.22 mmol, 3.0 equiv.), $Cu(OAc)_2{\cdot}H_2O$ (0.044 g, 0.22 mmol, 3.0 equiv.), and DMF (η = 0.28–1.2 μL/mg, Table S3). The resulting mixture was milled at 30 Hz for 90 min. The obtained solids were collected and washed by acetone (15 mL × 3) before the PXRD analysis for screening (Figure S5). Amounts of DMF ≥ 0.46 μL/mg produced similarly well-defined PXRD patterns.

Table S3. The amount of DMF additive was varied in attempts to synthesize the imine-linked **rht**-MOF using 90-min milling at 30 Hz. The conditions are summarized below, with corresponding PXRD patterns shown in Figure S5.

Entry	DMF additive (η) / μL/mg	DMF volume / μL
1	0.28	27
2	0.46	44
3	0.60	58
4	0.75	72
5	0.91	87
6	1.1	104

Synthesis of the imine-based hexacarboxylic ligand: 5,5',5''-((benzene-1,3,5-triyltris(methaneylylidene))tris(azaneylylidene))triisophthalic acid

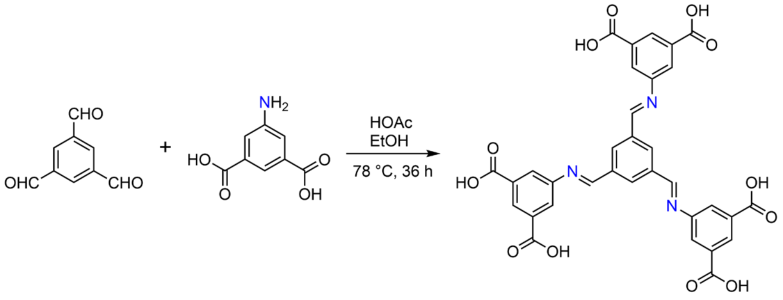

A 100-mL round bottom flask was charged with benzene-1,3,5-tricarbaldehyde (0.15 g, 0.093 mmol, 1.0 equiv.), 5-aminoisophthalic acid (0.50 g, 0.28 mmol, 3.0 equiv.), EtOH (50 mL), and acetic acid (3 drops). The resulting mixture was rigorously stirred under reflux for 36 h. The hot mixture with precipitation was filtered and washed with EtOH to yield the titled compound as a white solid (74% yield). Be cautious about rapid decomposition of the product in the air. ^{1}H NMR (δ, 23 °C, d_6-DMSO, Figure S6): 8.10 (d, *J* = 1.50 Hz, 6H), 8.39 (t, *J* = 1.20 Hz, 3H), 8.78 (s, 3H), 9.02 (s, 3H), 13.41 (br, 6H). High-resolution mass spectrometry (ESI, negative) data, calc $[C_{33}H_{19}N_3O_{12}]^{2-}$: 324.54899, expt m/z = 324.54938. IR (cm^{-1}, Figure S7): 1716 (s), 1629 (m), 1602 (s), 1585 (m), 1167 (m), 1456 (w), 1404 (w), 1362 (w), 1277 (m), 1235 (m), 1190 (m), 1125 (w), 986 (w), 960 (w), 913 (w), 760 (m), 680 (m), 666 (m).

Mechanochemical synthesis of the imine-linked rht-MOF using the pre-formed ligand

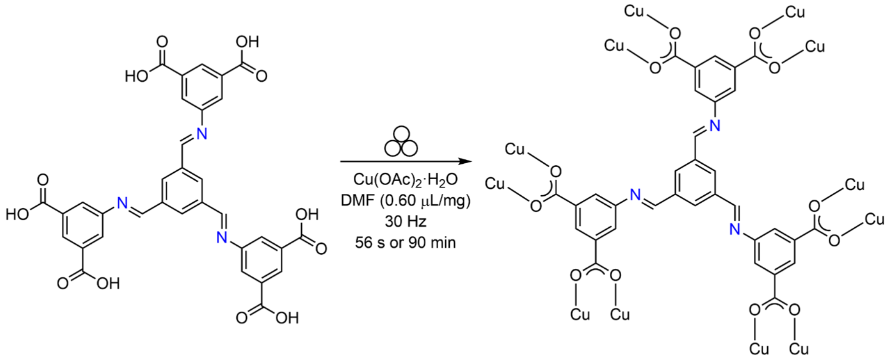

A 10-mL stainless steel grinding jar was charged with two stainless-steel grinding balls, the imine-based hexacarboxylic ligand (0.060 g, 0.093 mmol, 1.0 equiv.), $Cu(OAc)_2{\cdot}H_2O$ (0.055 g, 0.28 mmol, 3.0 equiv.), and DMF (η = 0.60 μL/mg, 69 μL). The resulting mixture was milled at 30 Hz for 56 s or 90 min. The obtained solids were collected and washed by acetone (15 mL × 3) before the PXRD analysis (Figure S8). Both reactions afforded the same desired MOF based on PXRD patterns. Further characterizations, including IR, N_2 adsorption analysis, and TGA, were collected with the 90-min product. IR (cm^{-1}, Figure S10a): 1707 (m), 1637 (m), 1558 (s), 1443 (s), 1412 (m), 1371 (s), 1311 (m), 11232 (w), 1220 (w), 1154 (w), 1107 (w), 1092 (w), 1003 (w), 966 (w), 910 (w), 776 (m), 375 (m), 688 (w), 531 (w), 479 (w). N_2 adsorption isotherm at 77 K, Figure S12b and Table S14. TGA, Figure S11.

Solvothermal synthesis of the imine-linked rht-MOF

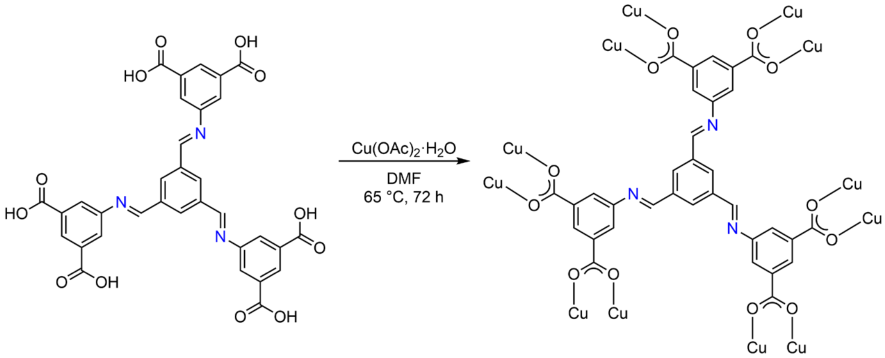

A 20-mL scintillation vial was charged with the imine-based hexacarboxylic ligand (0.016 g, 0.025 mmol, 1.0 equiv), $Cu(OAc)_2{\cdot}H_2O$ (0.020 g, 0.10 mmol, 4.0 equiv.), and DMF (2.0 mL) with or without and a chosen modulator (see Table S4). The resulting mixture was sonicated for 5 min and then placed in a preheated oven at 65 °C for 72 h. The resulting solids were collected and dried under reduced pressure for further characterization.

Based on PXRD analysis (Figure S9), the reaction outcomes are summarized in Table S4. In absence of an acid modulator (Entry 1), gel-like amorphous solids were obtained. In contrast, the presence of acetic acid (Entry 2) or conc. HNO_3 (Entry 3) afforded the desired **rht**-MOF, albeit in visibly low yields. Use of 1 M aqueous HNO_3 (Entry 4) led to mixed phases, which is attributed to the water-rich environment causing decomposition of the pre-formed imine ligand. Consequently, products from Entries 2 and 3 were selected for further characterization, including IR (Figure S10b), TGA (Figure S11), and N_2 adsorption analysis (Figure S12a and Table S14). Prior to gas adsorption analysis, the DMF saturated sample was exchanged with anhydrous dichloromethane for 3 days with the solvent replaced three times per day (15 mL each time).

Table S4. Different modulators were tested in the solvothermal synthesis of the imine-linked **rht**-MOF using the pre-synthesized hexacarboxylic ligand. The reactions are summarized below with corresponding PXRD patterns shown in Figures S9.

Entry	Modulator	Amount	Result
1	none	none	gel-like blue solids with poor crystallinity
2	acetic acid	129 µL	blue solids with matching PXRD patterns
3	conc. HNO_3	1 drop	green solids with matching PXRD patterns
4	1 M HNO_3	200 µL	green solids mixed with an impure phase

Screening of reaction time for the mechanochemical synthesis of imine-linked rht-MOF

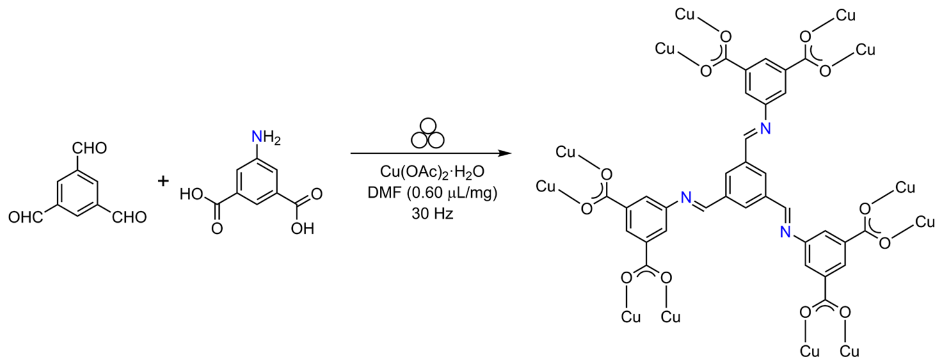

A 10-mL stainless steel grinding jar was charged with two stainless-steel grinding balls, benzene-1,3,5-tricarboxaldehyde (0.012 g, 0.074 mmol, 1.0 equiv.), 5-aminoisophthalic acid (0.040 g, 0.22 mmol, 3.0 equiv.), $Cu(OAc)_2{\cdot}H_2O$ (0.044 g, 0.22 mmol, 3.0 equiv.), and DMF (58 μL, η = 0.60 μL/mg). The resulting mixture was milled at 30 Hz for various durations (Table S5). The obtained solids were collected and washed by acetone (15 mL × 3) before the PXRD analysis (Figure S13). The 14-s and 28-s trials exhibit PXRD peaks of the $Cu(OAc)_2{\cdot}H_2O$ precursor, indicating an uncomplete reaction. The 56-s trial shows complete conversion.

Table S5. Milling time was varied in attempts to synthesize the imine-linked **rht**-MOF. The conditions are summarized below, with corresponding PXRD patterns shown in Figure S13. (*) denotes the sample showing PXRD peaks of the $Cu(OAc)_2{\cdot}H_2O$ precursor.

Entry	Time
1*	14 s
2*	28 s
3	56 s
4	1.85 min
5	3.75 min
6	7.5 min
7	15 min
8	30 min
9	60 min
10	90 min

Optimized mechanochemical synthesis of imine-linked rht-MOF

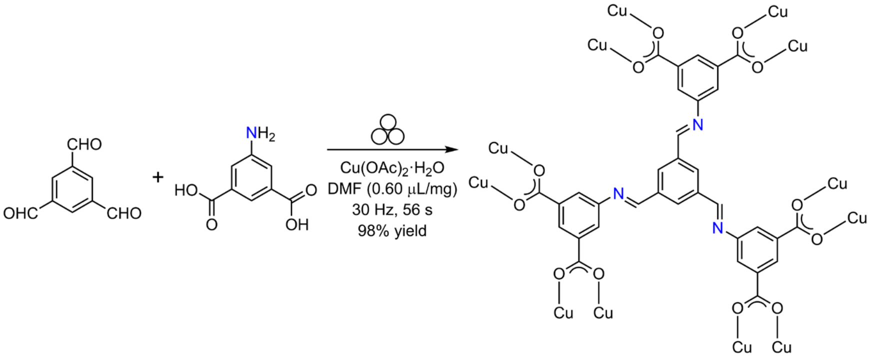

A 10-mL stainless steel grinding jar was charged with two stainless-steel grinding balls, benzene-1,3,5-tricarboxaldehyde (0.012 g, 0.074 mmol, 1.0 equiv.), 5-aminoisophthalic acid (0.040 g, 0.22 mmol, 3.0 equiv.), $Cu(OAc)_2{\cdot}H_2O$ (0.044 g, 0.22 mmol, 3.0 equiv.), and DMF (58 μL, η = 0.60 μL/mg). The resulting mixture was milled at 30 Hz for 56 seconds. The obtained solids were collected and washed by acetone (15 mL × 3). The solids were dried under reduced pressure to afford the desired imine-linked **rht**-MOF (0.060 g, 98% yield) as blue powder. Primary data are presented below: PXRD, Figures 3a and S13; IR (cm^{-1}, Figure S10a), 1698(m), 1629(s), 1559(s), 1445(m), 1411(m), 1369(s), 1144(w), 1110(w), 1002(w), 964(w), 909(w), 778(m), 729(m), 681(m); TGA, Figure S11; N_2 adsorption isotherm at 77 K, Figure 3b.

Mechanochemical synthesis of the expanded imine-linked rht-MOF

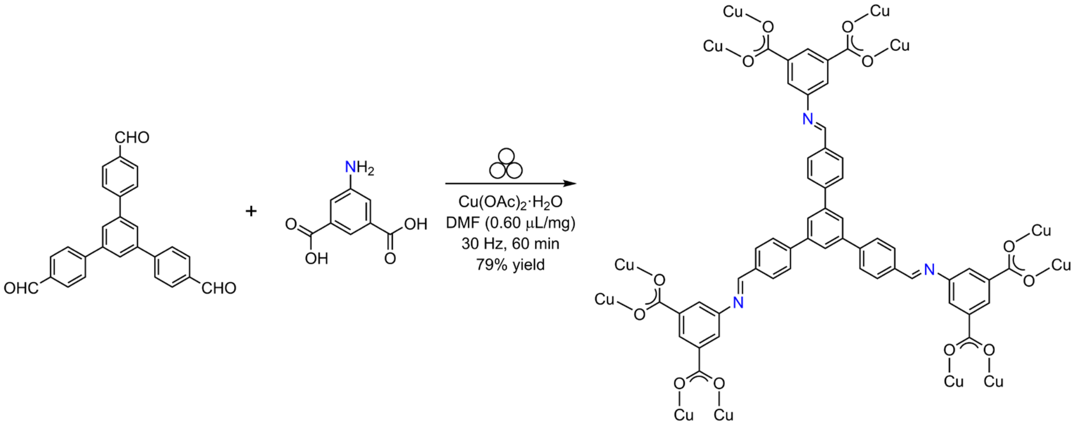

A 10-mL stainless steel grinding jar was charged with two stainless steel grinding balls, 5-aminoisophthalic acid (0.050 g, 0.28 mmol, 3.0 equiv.), 5'-(4-formylphenyl)-[1,1':3',1''-terphenyl]-4,4''-dicarbaldehyde (0.036 g, 0.092 mmol, 1.0 equiv.), $Cu(OAc)_2·H_2O$ (0.055 g, 0.28 mmol, 3.0 equiv.), and DMF (43 μL, η = 0.30 μL/mg). The resulting mixture was milled at 30 Hz for 60 min. The obtained solids were collected and washed by acetone (15 mL × 3). The solids were dried under reduced pressure to afford the expanded imine-linked **rht**-MOF. (0.078 g, 79% yield) as a dark green powder. Primary data are presented below: PXRD, Figures 4 and S14; IR (cm^{-1}, Figure S15), 1691(w), 1632(m), 1576(s), 1476(w), 1442(w), 1372(s), 1214(w), 1172(w), 1099(w), 1002(w), 961(w), 891(w), 854(w), 823(w), 771(m), 726(m); TGA, Figure S16; N_2 adsorption isotherm at 77 K, Figure S17 and Table S15.

Table S6. Experimental parameters, including milling time and the amount of DMF additive, were varied in attempts to synthesize the expanded imine-linked **rht**-MOF. The reaction conditions are summarized below, with corresponding PXRD patterns shown in Figures S14.

Entry	Time / min	DMF additive (η / μL/mg, volume / μL)
1	60	0.30, 43
2	60	0.45, 64
3	60	0.60, 85
4	60	0.75, 106
5	60	0.91, 128
6	90	0.60, 85

Synthesis of the expanded imine-based hexacarboxylic ligand

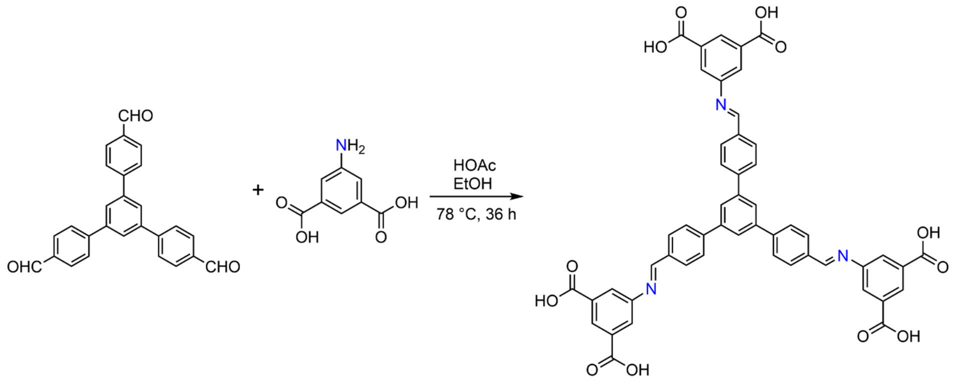

A 100-mL round bottom flask was charged with 5'-(4-formylphenyl)-[1,1':3',1''-terphenyl]-4,4''-dicarbaldehyde (0.36 g, 0.093 mmol, 1.0 equiv.), 5-aminoisophthalic acid (0.50 g, 0.28 mmol, 3.0 equiv.), EtOH (50 mL), and acetic acid (3 drops). The resulting mixture was rigorously stirred under reflux for 36 h. The hot mixture with precipitation was filtered and washed with EtOH to yield the product as a pale-yellow solid (12% yield). Be cautious about rapid decomposition of the product in the air. ^{1}H NMR (δ, 23 °C, d_6-DMSO, Figure S18): 8.02 (d, *J* = 1.50 Hz, 6H), 8.15 (m, 15H), 8.37 (t, *J* = 1.20 Hz, 3H), 8.87 (s, 3H). High-resolution mass spectrometry (ESI, negative) data, calc $[C_{51}H_{31}N_3O_{12}]^{2-}$: 438.59594, expt m/z = 438.59634. IR (cm^{-1}, Figure S15): 1699 (s), 1626 (m), 1599 (s), 1590 (s), 1563 (m), 1514 (w), 1449 (m), 1411 (m), 1397 (m), 1301 (m), 1281 (m), 1229 (m), 1280 (m), 1109 (w), 1015 (w), 997 (w), 965 (m), 909 (m), 871 (w), 826 (m), 811 (m), 760 (m), 739 (w), 728 (w), 663 (m), 607 (w), 574 (w), 563 (w), 518 (w).

Mechanochemical synthesis of the expanded imine-linked rht-MOF using the pre-formed imine ligand

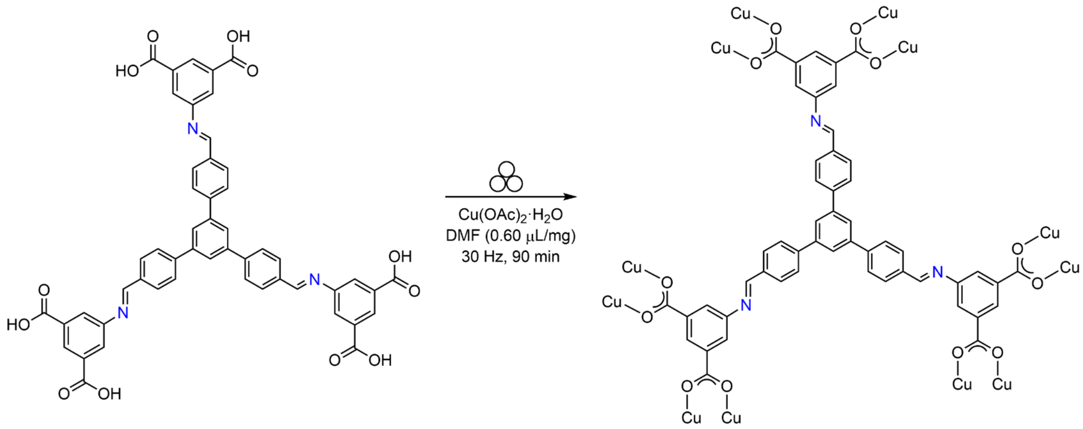

A 10-mL stainless steel grinding jar was charged with two stainless-steel grinding balls, the expanded imine-based hexacarboxylic ligand (0.081 g, 0.093 mmol, 1.0 equiv.), $Cu(OAc)_2 \cdot H_2O$ (0.055 g, 0.28 mmol, 3.0 equiv.), and DMF (82 μL, η = 0.60 μL/mg). The resulting mixture was milled at 30 Hz for 90 min. The obtained solids were collected and washed by acetone (15 mL × 3) before the PXRD analysis (Figure S19b). This mechanochemical reaction afforded the desired expanded imine-based **rht**-MOF, indicating the imine bond formation and cleavage is reversible under the mechanochemical conditions. IR (cm^{-1}, Figure S20a): 1706 (m), 1631 (s), 1602 (s), 1581 (s), 1563 (s), 1444 (m), 1414 (s), 1368 (s), 1303 (m), 1216 (m), 1173 (m), 1106 (w), 1090 (w), 1016 (w), 1001 (w), 966 (w), 912 (w), 869 (w), 853 (w), 825 (w), 795 (w), 772 (m), 730 (m), 682 (w), 625 (w), 608 (w), 530 (w), 483 (w). N_2 adsorption isotherm at 77 K, Figure S21 and Table S16. TGA, Figure S16.

Solvothermal synthesis of the expanded imine-linked rht-MOF

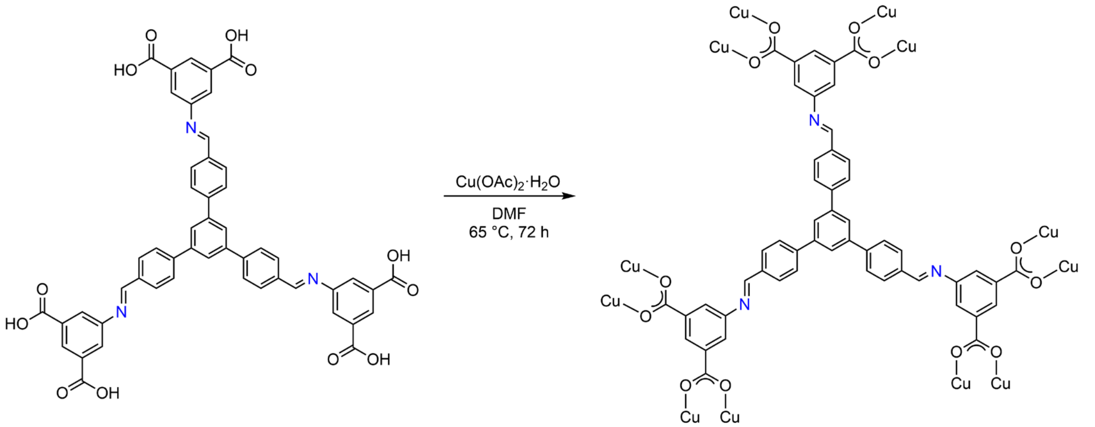

A 20-mL scintillation vial was charged with the expanded imine-based hexacarboxylic ligand (0.022 g, 0.025 mmol, 1.0 equiv), $Cu(OAc)_2 \cdot H_2O$ (0.020 g, 0.10 mmol, 4.0 equiv.), and DMF (2.0 mL) with or without a chosen modulator (see Table S7). The resulting mixture was sonicated for 5 min and then placed in a preheated oven at 65 °C for 72 h. The resulting solids were collected and dried under reduced pressure for further characterization.

PXRD analysis (Figure S19a) reveals that, in the absence of an acid modulator (Entry 1) as well as in the presence of acetic acid (Entry 2) or concentrated HNO_3 (Entry 3), the desired rht-MOF phase was obtained, albeit in visibly low yields. In contrast, use of 1 M aqueous HNO_3 (Entry 4) led to a completely different phase, which is attributed to the water-rich environment causing decomposition of the preformed imine ligand. Owing to the poor crystallinity observed for the material from Entry 1, subsequent studies focused on products from Entries 2 and 3, which were further characterized by IR (Figure 20b) and TGA (Figure S16). In addition, N_2 adsorption isotherms at 77 K (Figure S21 and Table S16) were collected for the MOF obtained from Entry 2, which has relatively better crystallinity. Prior to gas adsorption analysis, the DMF saturated sample was exchanged with anhydrous dichloromethane for 3 days with the solvent replaced three times per day (15 mL each time).

Table S7. Different modulators were tested in the solvothermal synthesis of the expanded imine-linked **rht**-MOF using the pre-synthesized hexacarboxylic ligand. The reactions are summarized below with corresponding PXRD patterns shown in Figures S19a.

Entry	Modulator	Amount
1	none	none
2	acetic acid	129 μL
3	conc. HNO_3	1 drop
4	1 M HNO_3	200 μL

Mechanochemical synthesis of the imine-linked pto-MOF

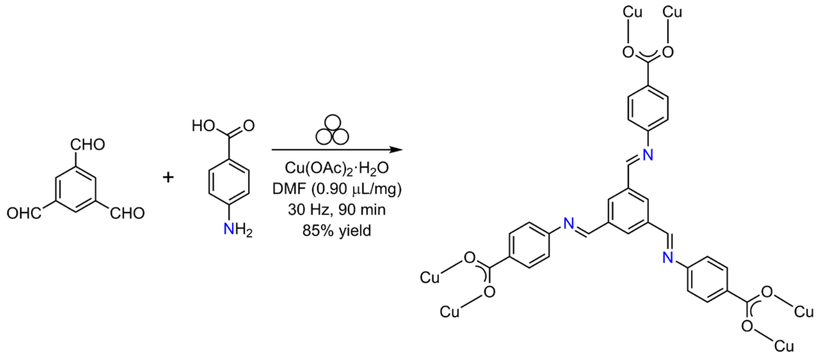

A 10-mL stainless steel grinding jar was charged with two stainless steel grinding balls, benzene-1,3,5-tricarboxaldehyde (0.025 g, 0.15 mmol, 1.0 equiv.), 4-aminobenzoic acid (0.062 g, 0.46 mmol, 3.0 equiv.), $Cu(OAc)_2{\cdot}H_2O$ (0.046 g, 0.23 mmol, 1.5 equiv.), and DMF (120 μL, η = 0.90 μL/mg). The resulting mixture was milled at 30 Hz for 90 min. The obtained solids were collected and washed by acetone (15 mL × 3). The solids were dried under reduced pressure to afford the imine-linked **pto**-topology MOF (0.080 g, 85% yield) as a blue powder. Primary data are presented below: PXRD, Figures 5a, S22, and S23; IR (cm^{-1}, Figure S24), 1698(w), 1598(s), 1541(w), 1397(s), 1253(m), 1173(m), 1142(m), 1105(w), 1014(w), 973(w), 858(w), 785(m), 685(m); TGA, Figure S25; N_2 adsorption isotherm at 77 K, Figure 5b.

Table S8. Experimental parameters, including milling time and the amount of DMF additive, were varied in attempts to synthesize the imine-linked **pto**-MOF. The reaction conditions are summarized below, with corresponding PXRD patterns shown in Figures S22–S23.

Entry	Time / min	DMF additive (η / μL/mg, volume / μL)
1	90	0.15, 20
2	90	0.30, 40
3	90	0.45, 60
4	90	0.60, 80
5	90	0.75, 100
6	90	0.90, 120
7	75	0.90, 120
8	60	0.90, 120
9	45	0.90, 120
10	30	0.90, 120
11	23	0.90, 120
12	15	0.90, 120
13	7.5	0.90, 120

Synthesis of the imine-based tricarboxylic ligand: 4,4',4''-((benzene-1,3,5-triyltris(methaneylylidene))tris(azaneylylidene))tribenzoic acid

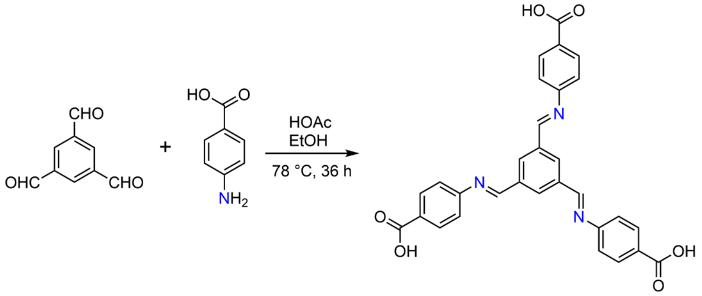

A 100-mL round bottom flask was charged with benzene-1,3,5-tricarbaldehyde (0.15 g, 0.093 mmol, 1.0 equiv.), 4-aminobenzoic acid (0.38 g, 0.28 mmol, 3.0 equiv.), EtOH (50 mL), and acetic acid (3 drops). The resulting mixture was rigorously stirred under reflux for 36 h. The solvent was removed under reduced pressure, and the obtained solids were filtered and washed with EtOH to yield the titled compound as a white solid (34% yield). Be cautious about rapid decomposition of the product in the air. ^{1}H NMR (δ, 23 °C, d_6-DMSO, Figure S26): 7.42 (d, J = 8.40 Hz, 6H), 8.02 (d, J = 8.40 Hz, 6H), 8.72 (s, 3H), 8.89 (s, 3H), 12.93 (br, 3H). High-resolution mass spectrometry (ESI, negative) data, calc $[C_{30}H_{20}N_3O_6]^-$: 518.13576, expt m/z = 518.13574. IR (cm^{-1}, Figure S24): 1699 (s), 1629 (m), 1590 (s), 1521 (w), 1503 (w), 1424 (s), 1369 (w), 1317 (m), 1288 (s), 1241 (s), 1169 (s), 1110 (w), 1045 (w), 1013 (w), 981 (w), 921 (w), 882 (w), 865 (m), 850 (m), 770 (s), 699 (m), 671 (m), 634 (w), 609 (w), 685 (w), 548 (m), 513 (w), 463 (w).

Mechanochemical synthesis of the imine-linked pto-MOF using the pre-formed ligand

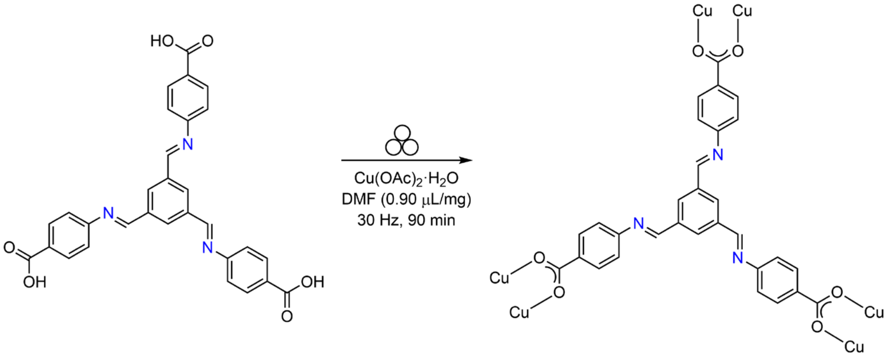

A 10-mL stainless steel grinding jar was charged with two stainless-steel grinding balls, the imine-based tricarboxylic ligand (0.080 g, 0.015 mmol, 1.0 equiv.), $Cu(OAc)_2{\cdot}H_2O$ (0.046 g, 0.23 mmol, 1.5 equiv.), and DMF (113 µL, η = 0.90 µL/mg). The resulting mixture was milled at 30 Hz for 90 min. The obtained solids were collected and washed by acetone (15 mL × 3) before the PXRD analysis (Figure S27). This mechanochemical reaction afforded the desired imine-linked **pto**-MOF, indicating the imine bond formation and cleavage is reversible under the mechanochemical conditions. IR (cm^{-1}, Figure S24): 1700 (m), 1626 (m), 1594 (s), 1537 (s), 1500 (m), 1400 (s), 1251 (w), 1219 (w), 1168 (m), 1143 (m), 1102 (w), 1012 (w), 974 (w), 863 (m), 783 (m), 702 (m), 683 (m), 638 (w), 604 (w), 525 (w), 463 (w). N_2 adsorption isotherm at 77 K, Figure S28 and Table S17. TGA, Figure S25.

Solvothermal synthesis attempts of the imine-linked pto-MOF

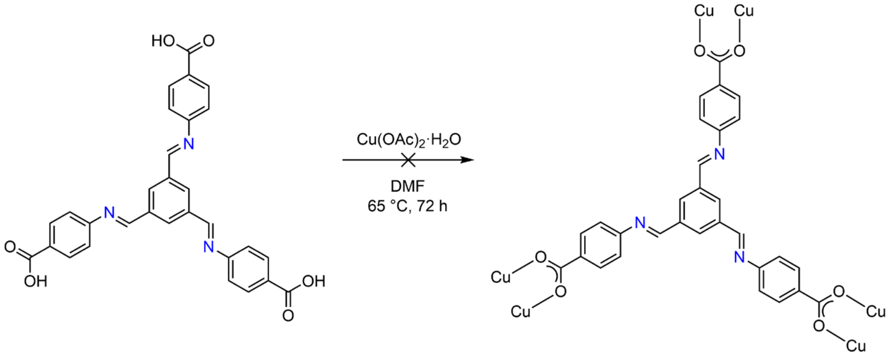

A 20-mL scintillation vial was charged with the imine-based tricarboxylic ligand (0.013 g, 0.025 mmol, 1.0 equiv), $Cu(OAc)_2 \cdot H_2O$ (0.020 g, 0.10 mmol, 4.0 equiv.), and DMF (2.0 mL) with or without a chosen modulator (see Table S9). The resulting mixture was sonicated for 5 min and then placed in a preheated oven at 65 °C for 72 h. In all four attempts, a clear dark turquoise solution was observed with no precipitation. This outcome is likely due to decomposition of the imine-linked ligands under solvothermal conditions. Consequently, no desired MOF could be obtained under these conditions.

Table S9. Different modulators were tested in the solvothermal synthesis of the imine-linked **pto**-MOF using the pre-synthesized tricarboxylic ligand.

Entry	Modulator	Amount
1	none	none
2	acetic acid	129 µL
3	conc. HNO_3	1 drop
4	1 M HNO_3	200 µL

Mechanochemical attempts to synthesize the amide-linked pto-MOF

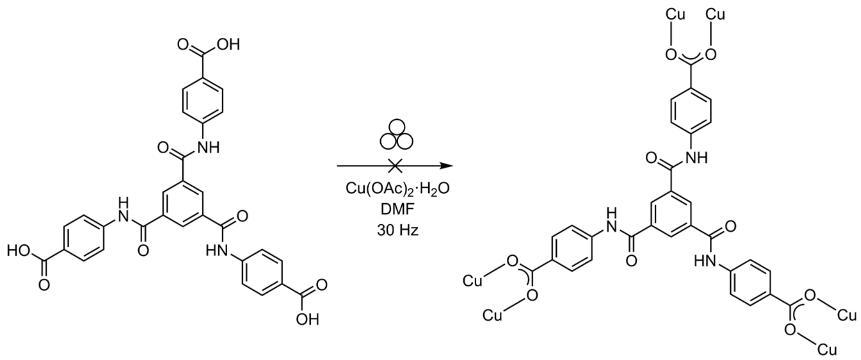

A 10-mL stainless steel grinding jar was charged with two stainless-steel grinding balls, 4,4',4''-((benzene-1,3,5-tricarbonyl)tris(azanediyl))tribenzoic acid (0.053 g, 0.093 mmol, 1.0 equiv.), $Cu(OAc)_2 \cdot H_2O$ (0.028 g, 0.14 mmol, 1.5 equiv.), and DMF (η = 0.60 µL/mg, or 0.90 µL/mg). The resulting mixture was milled at 30 Hz for various durations (Table S10). The obtained solids were collected and washed with acetone (15 mL × 3) before PXRD analysis (Figure S29). In addition to DMF, we also examined MeOH and H_2O (PXRD patterns shown in Figure S30, η = 0.60µL/mg, 48 µL, t = 90 min) in attempts to synthesize the amide-linked **pto**-MOF mechanochemically, without success.

Table S10. Experimental parameters, including milling time, the amount of DMF additive, and the type of liquid additive, were varied in attempts to synthesize the amide-linked **pto**-MOF. The reaction conditions are summarized below, with corresponding PXRD patterns shown in Figures S29–S30.

Entry	Time / min	liquid additive (η / µL/mg, volume / µL)
1	90	DMF, 0.60, 48
2	90	DMF, 0.90, 72
3	60	DMF, 0.60, 48
4	60	DMF, 0.90, 72
5	45	DMF, 0.90, 72
6	90	MeOH, 0.60, 48
7	90	H_2O, 0.60, 48

Mechanochemical attempts to synthesize the alkyne-linked pto-MOF

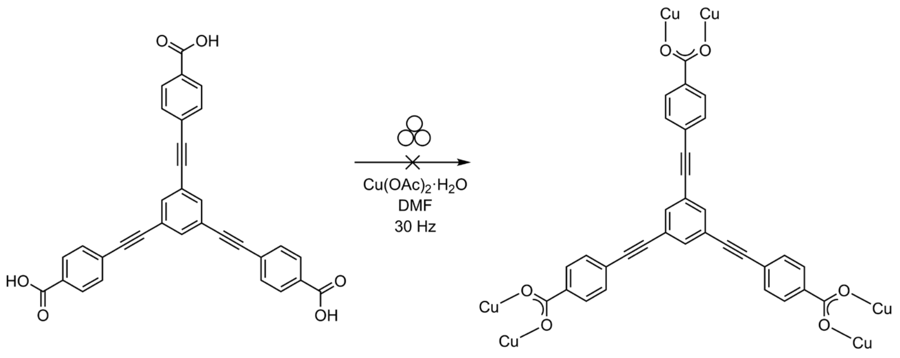

A 10-mL stainless steel grinding jar was charged with two stainless-steel grinding balls, 4,4',4''-(benzene-1,3,5-triyltris(ethyne-2,1-diyl))tribenzoic acid (0.047 g, 0.093 mmol, 1.0 equiv), $Cu(OAc)_2 \cdot H_2O$ (0.028 g, 0.14 mmol, 1.5 equiv), and DMF (η = 0.31, 0.60, 0.75 or 0.91 µL/mg). The resulting mixture was milled at 30 Hz for various durations (Table S11). The obtained solids were collected and washed with acetone (15 mL × 3) before PXRD analysis (Figures S31–S32).

Table S11. Experimental parameters, including milling time and the amount of DMF as the liquid additive, were varied in attempts to synthesize the alkyne-linked **pto**-MOF. The reaction conditions are summarized below, and corresponding PXRD patterns are shown in Figures S31–S32.

Entry	Time / min	DMF additive (η / µL/mg, volume / µL)
1	7.5	0.75, 56
2	15	0.75, 56
3	30	0.75, 56
4	60	0.75, 56
5	90	0.75, 56
6	90	0.91, 68
7	90	0.60, 45
8	90	0.31, 23

Mechanochemical synthesis of MOF-14

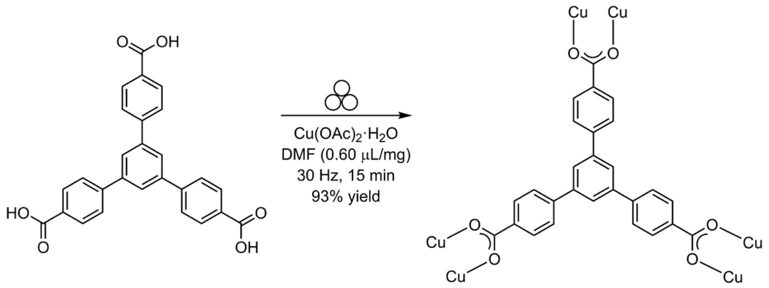

The mechanochemical synthesis of MOF-14 has been previously reported.[6, 7] We actively investigated experimental parameters (Table S12), including milling time and the amount of EtOH additive in the mechanochemical reaction. Below is our modified procedure, which affords MOF-14 with an improved BET surface area. A 10-mL stainless steel grinding jar was charged with 2 stainless steel grinding balls, 1,3,5-tris(4-carboxyphenyl)benzene (H_3btb, 0.076 g, 0.17 mmol, 1.0 equiv.), $Cu(OAc)_2 \cdot H_2O$ (0.052 g, 0.26 mmol, 1.5 equiv.), and EtOH (77 μL, η = 0.60 μL/mg). The resulting mixture was milled at 30 Hz for 15 min. The obtained solids were collected and washed by acetone (15 mL × 3). The solids were dried under reduced pressure to afford MOF-14 (0.085 g, 93% yield) as a blue powder. Primary data are presented below: PXRD, Figures S33-S34; IR (cm^{-1}, Figure S35), 1695(w), 1606(m), 1583(m), 1532(m), 1397(s), 1265(w), 1241(w), 1183(w), 1110(w), 1016(w), 845(m), 807(w), 772(s), 703(w), 671(w); TGA, Figure S36; N_2 adsorption isotherm at 77 K, Figure S37 and Table S18.

Table S12. Experimental parameters, including milling time and the amount of EtOH additive, were varied in attempts to synthesize the **pto**-topology MOF-14. The reaction conditions are summarized below, with corresponding PXRD patterns shown in Figures S33–S34.

Entry	Time / min	EtOH additive (η / μL/mg, volume / μL)
1	90	0.60, 77
2	75	0.60, 77
3	60	0.60, 77
4	45	0.60, 77
5	30	0.60, 77
6	15	0.60, 77
7	45	0.45, 58
8	45	0.30, 38
9	45	0.90, 115
10	45	1.2, 153

C. Supporting Data

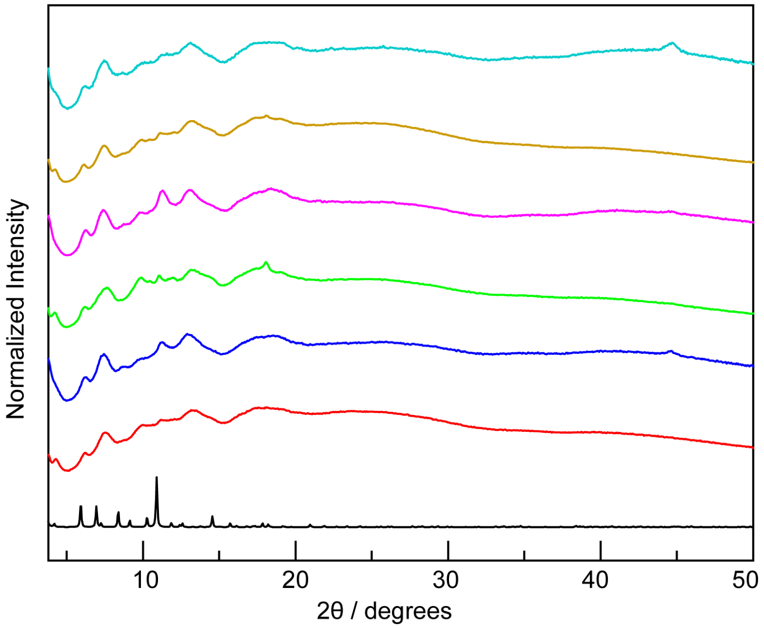

Figure S1. PXRD patterns were collected from experiments varying milling time and the amount of DMF additive in the mechanochemical synthesis of ether-linked **rht**-MOF: —, Simulated; —, η = 0.60 μL/mg, t = 60 min; —, η = 0.90 μL/mg, t = 60 min; —, η = 0.60 μL/mg, t = 90 min; —, η = 0.90 μL/mg, t = 90 min; —, η = 0.60 μL/mg, t = 120 min; —, η = 0.90 μL/mg, t = 120 min. None of these typical milling conditions yielded the desired phase.

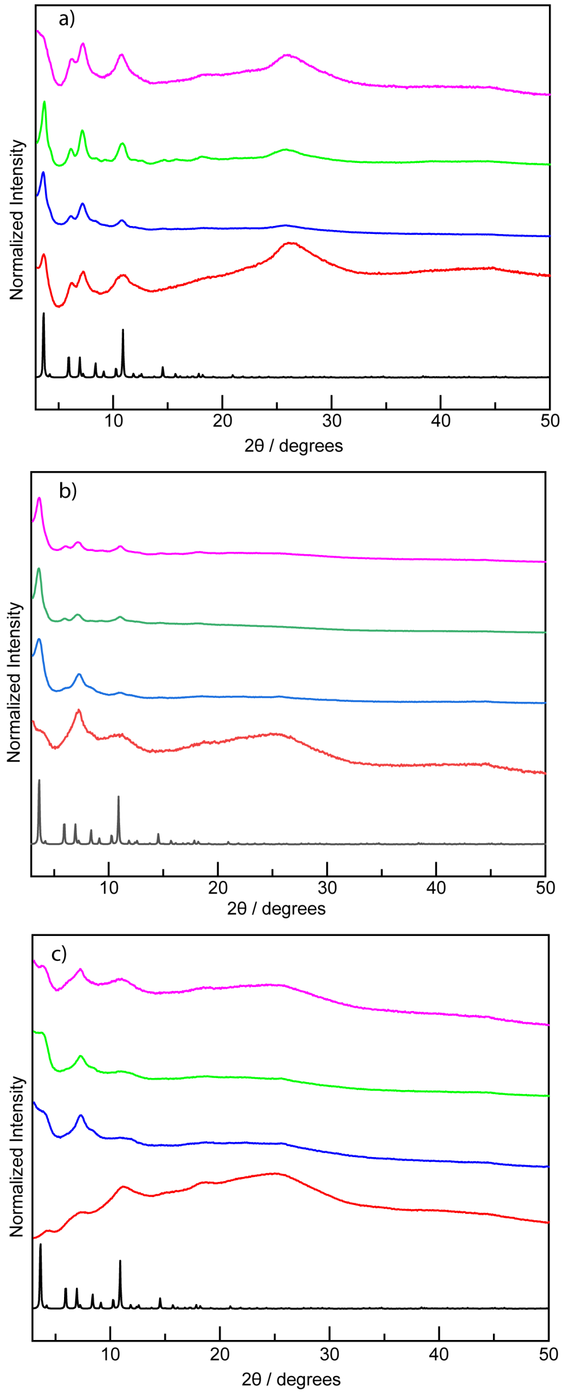

Figure S2. (a) PXRD patterns were collected from experiments with DMF additive (η = 0.60 μL/mg, 73 μL) at varying milling times for the synthesis of the amide-linked **rht**-MOF: —, simulated; —, *t* = 20 min; —, *t* = 60 min; —, *t* = 90 min; —, *t* = 120 min. (b) PXRD patterns were collected from experiments with DMF additive (η = 0.90 μL/mg, 109 μL) at varying milling times for the synthesis of the amide-linked **rht**-MOF: —, simulated; —, *t* = 20 min; —, *t* = 60 min; —, *t* = 90 min; —, *t* = 120 min. (c) PXRD patterns were collected from experiments with DMF additive (η = 1.2 μL/mg, 145 μL) at varying milling times for the synthesis of the amide-linked **rht**-MOF: —, simulated; —, *t* = 20 min; —, *t* = 60 min; —, *t* = 90 min; —, *t* = 120 min.

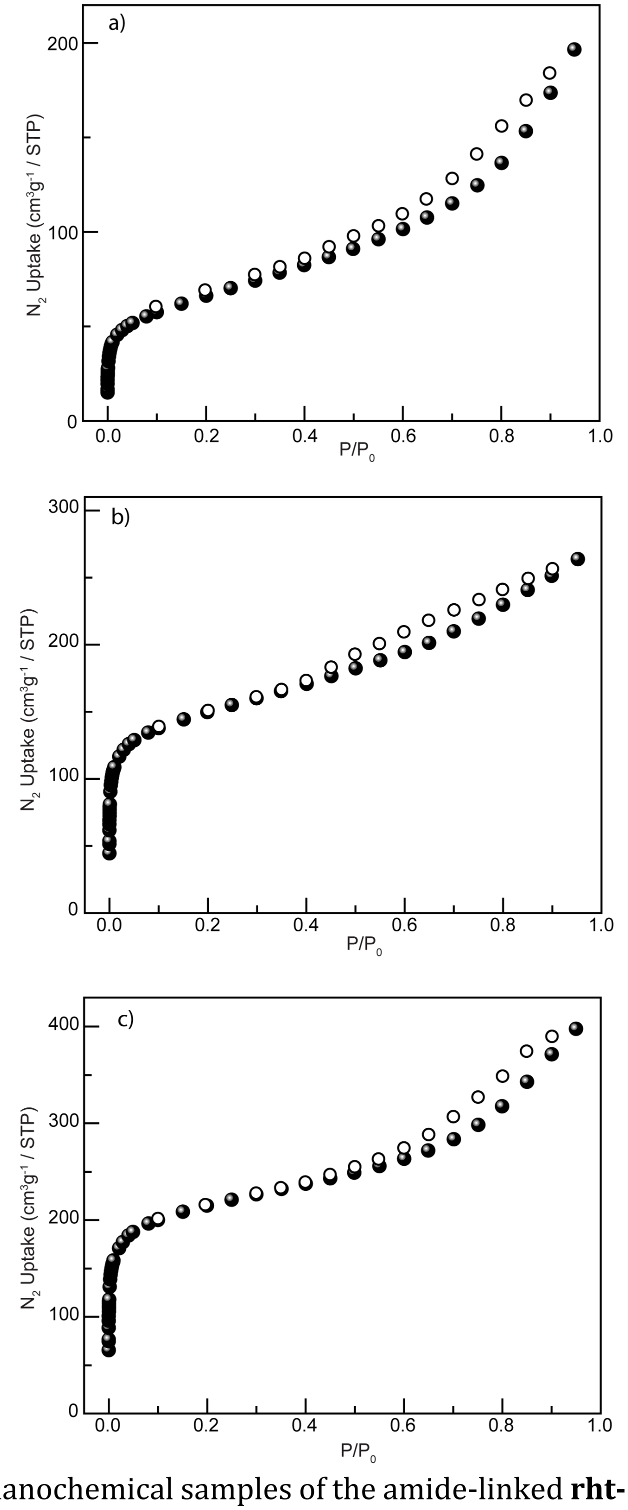

Figure S3. Three mechanochemical samples of the amide-linked **rht**-MOF with well-defined PXRD patterns were selected for N_2 adsorption analysis at 77 K. (a) N_2 adsorption isotherm (adsorption (●), desorption (○)) for the sample prepared with DMF (η = 0.60 μL/mg, 73 μL) and milled for 60 min (b) N_2 adsorption isotherm (adsorption (●), desorption (○)) for the sample prepared with DMF (η = 0.90 μL/mg, 109 μL) and milled for 90 min. (c) N_2 adsorption isotherms (adsorption (●), desorption (○)) for the sample with DMF (η = 0.90 μL/mg, 109 μL) and milled for 120 min.

Table S13. Surface area values of the mechanochemically obtained amide-linked **rht**-MOF were determined from N_2 adsorption isotherms measured at 77 K.

Time / min	DMF additive (η / μL/mg, volume / μL)	BET surface area / m^2/g (P/P_0 = 0.007-0.03)	Langmuir surface area / m^2/g (P/P_0 = 0.007-0.03)
60	0.60, 73	215	224
90	0.90, 109	373	389
120	0.90, 109	1069	1112

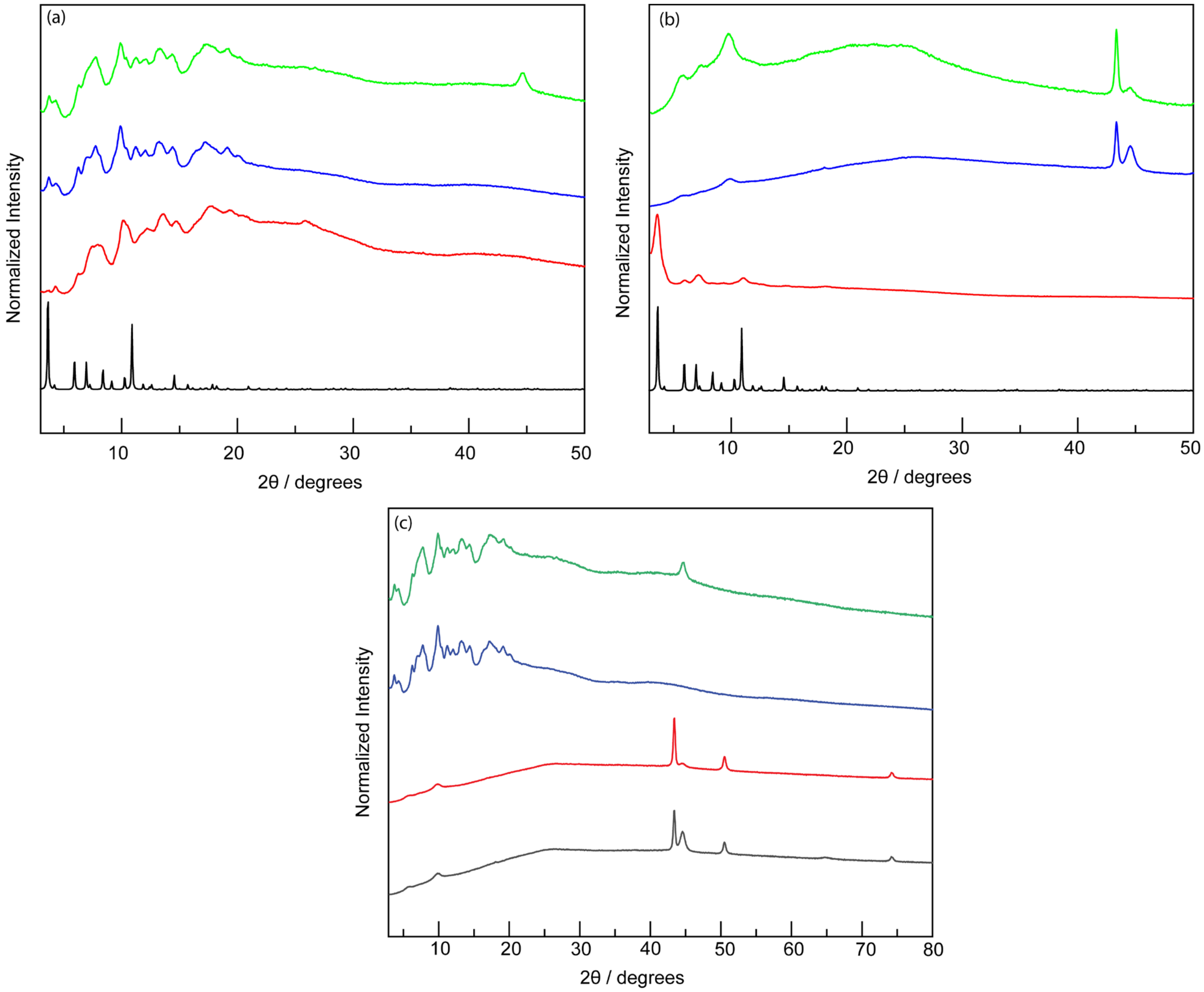

Figure S4. (a) PXRD patterns were collected from mechanochemical attempts to synthesize the ether-linked **rht**-MOF using 120 min milling at 30 Hz in the presence of DMF (η = 0.90 µL/mg) at different milling temperatures: —, simulated; —, room temperature; —, 80 °C; —, 120 °C. (b) PXRD patterns from analogous mechanochemical attempts to synthesize the amide-linked rht-MOF under identical conditions (120 min milling, 30 Hz, η = 0.90 µL/mg DMF): —, simulated; —, room temperature; —, 80 °C; —, 120 °C. In both systems, elevated milling temperatures did not yield the desired phase or improve upon the room-temperature trial; instead, reflections corresponding to elemental Cu were observed in the case of the amide reactions. (c) PXRD patterns were collected over a 2θ range of 3–80°: —, amide linker at 80 °C; —, amide linker at 120 °C; —, ether linker at 80 °C; —, ether linker at 120 °C.

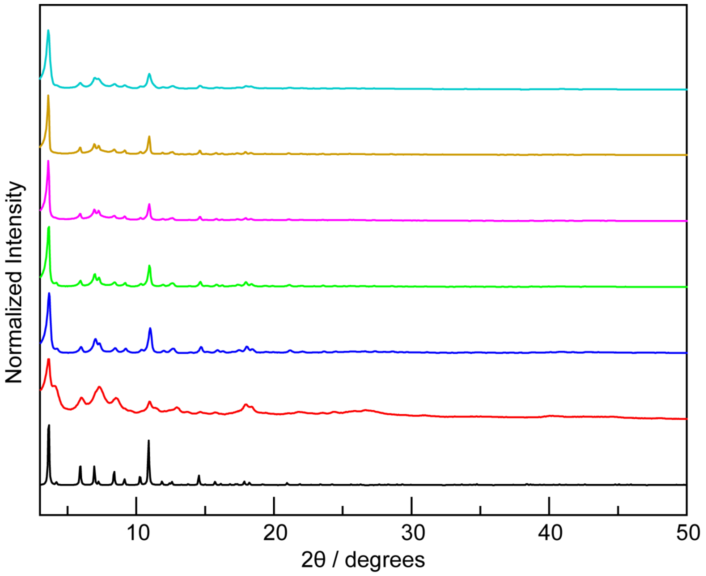

Figure S5. PXRD patterns were collected from experiments varying the amount of DMF additive in the mechanochemical synthesis of imine-linked **rht**-MOF using 90-min milling at 30 Hz: —, simulated; —, η = 0.28 μL/mg; —, η = 0.46 μL/mg; —, η = 0.60 μL/mg; —, η = 0.75 μL/mg; —, η = 0.91 μL/mg; —, η = 1.1 μL/mg. Amounts of DMF ≥ 0.46 μL/mg produced similarly well-defined PXRD patterns.

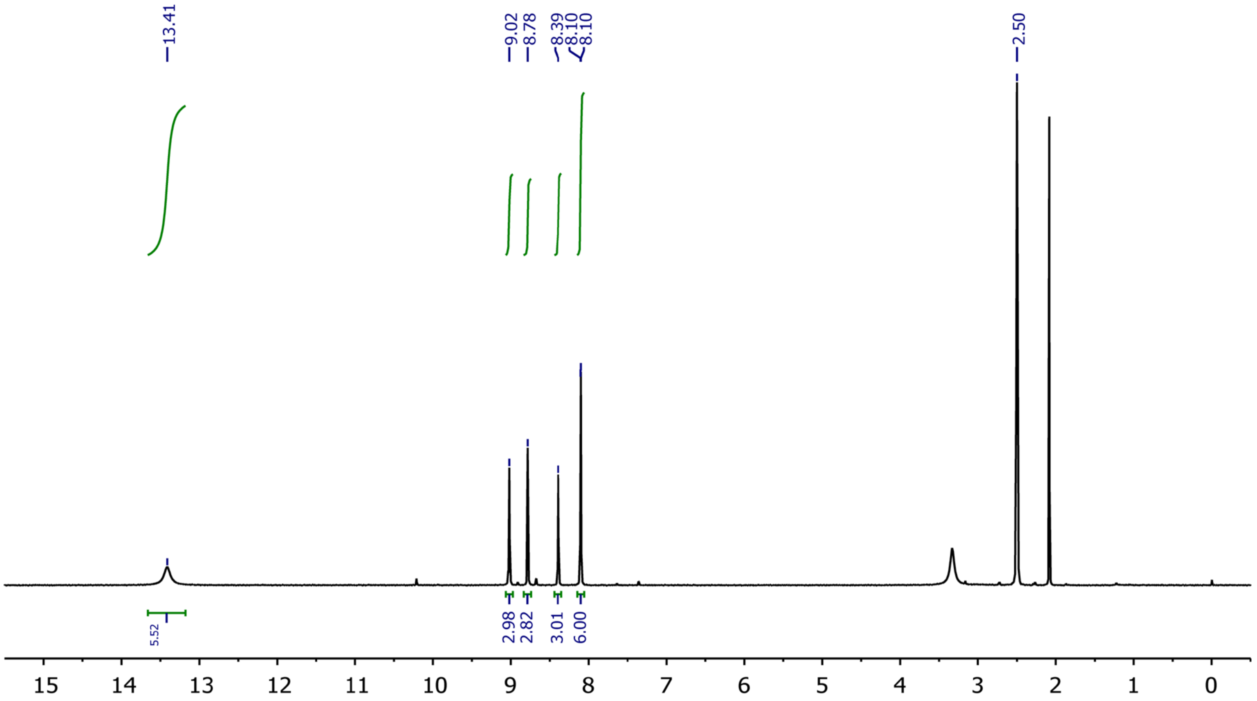

Figure S6. ^{1}H NMR spectrum of 5,5',5''-((benzene-1,3,5-triyltris(methaneylylidene))tris(azaneylylidene))triisophthalic acid (the imine-based hexacarboxylic ligand) obtained by the solution synthesis was acquired in d_6-DMSO at 23 °C.

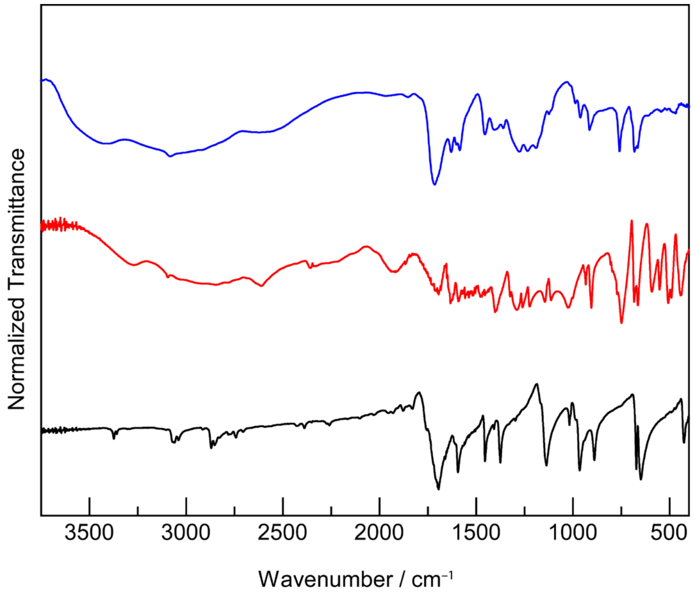

Figure S7. IR spectra of benzene-1,3,5-tricarbaldehyde (—), 5-aminoisophthalic acid (—), and the preformed imine-based hexacarboxylic ligand (5,5',5''-((benzene-1,3,5-triyltris(methaneylylidene))tris(azaneylylidene))triisophthalic acid, —).

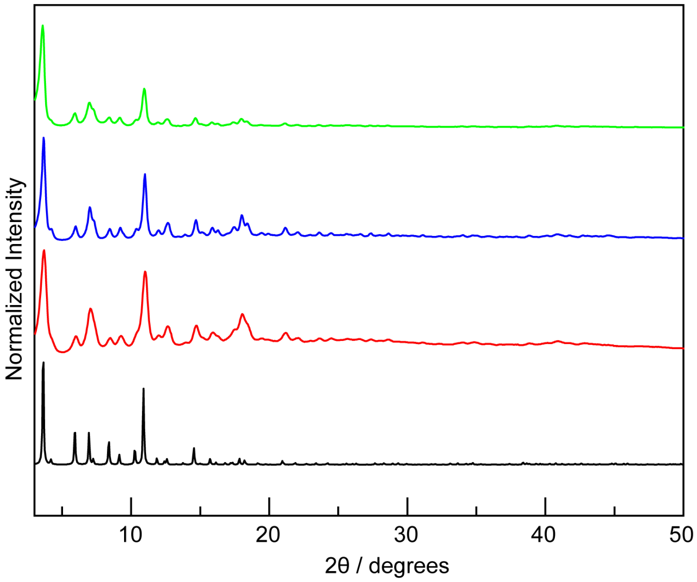

Figure S8. PXRD patterns were collected from mechanochemical syntheses of the imine-linked **rht**-MOF using the pre-synthesized imine-based hexacarboxylic ligand, conducted with 56 s or 90 min milling at 30 Hz in the presence of DMF (η = 0.60 μL/mg): —, simulated; —, 56 s; —, 90 min. Both reactions afforded the desired imine-linked **rht**-MOF and are consistent with the one-pot cascade reaction (—, 90 min) using aldehyde and primary amine precursors, highlighting that imine bond formation and cleavage are reversible under the applied mechanochemical conditions.

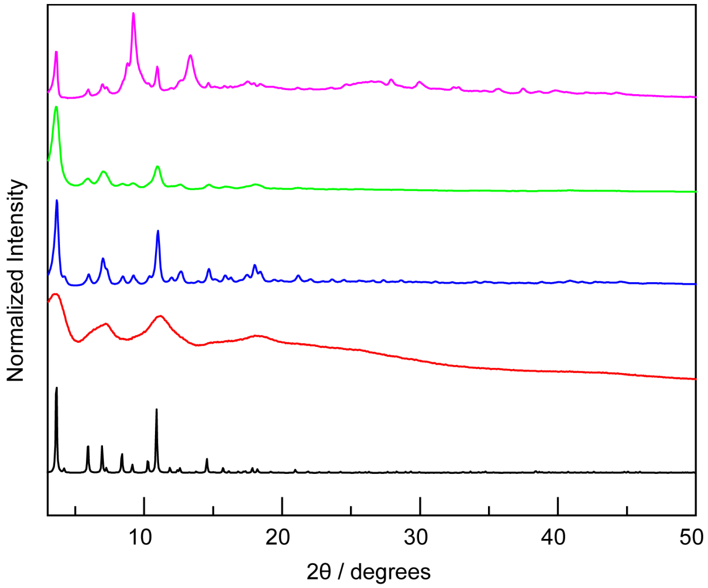

Figure S9. PXRD patterns were collected from solvothermal syntheses of the imine-linked **rht**-MOF using the pre-synthesized imine-based hexacarboxylic ligand, conducted at 65 °C for 72 h: —, simulated; —, without an acid modulator; —, HOAc; —, conc. HNO_3; —, 1 M aqueous HNO_3.

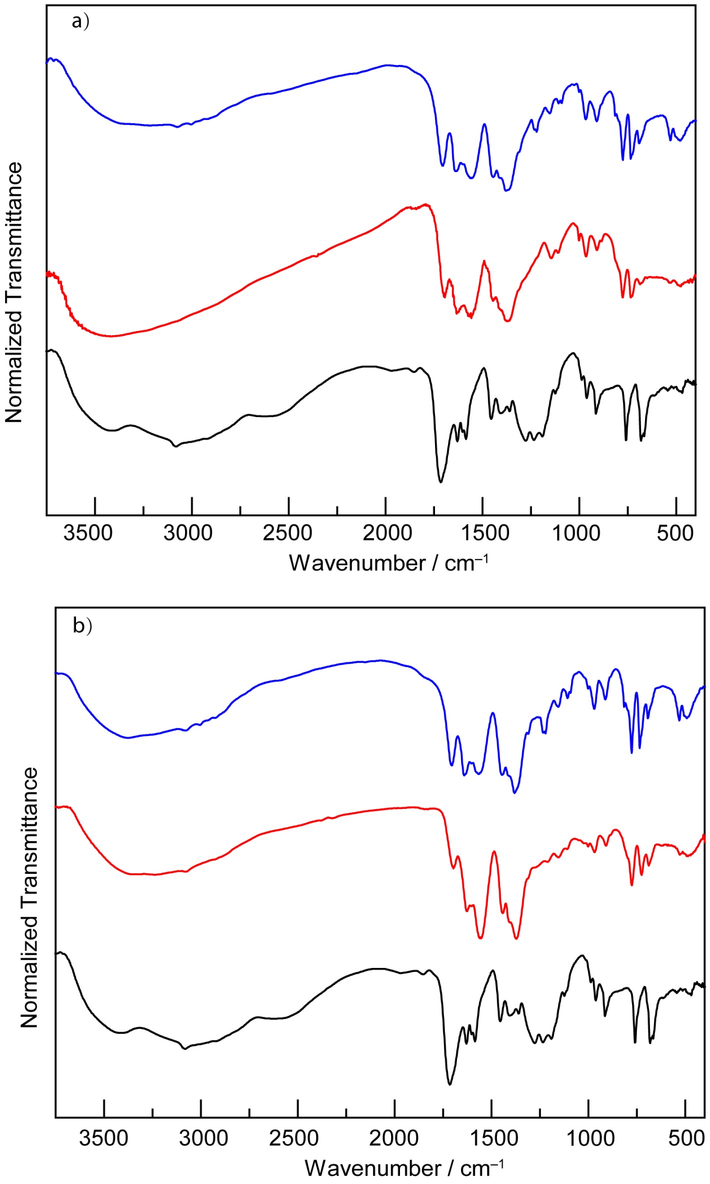

Figure S10. (a) IR spectra were collected from the preformed imine hexacarboxylic ligand (—) and the imine-linked **rht**-MOF obtained by tandem mechanochemical synthesis (—) and by mechanochemical synthesis using the preform ligand (—). (b) IR spectra were collected from the imine-linked **rht**-MOF obtained by solvothermal synthesis modulated with HOAc (—) and conc. HNO_3 (—), compared to the preformed imine ligand (—). The appearance of coordinated C=O stretch observed at 1371 cm^{-1} in the **rht**-MOF indicates the formation of dative metal–ligand bonds.

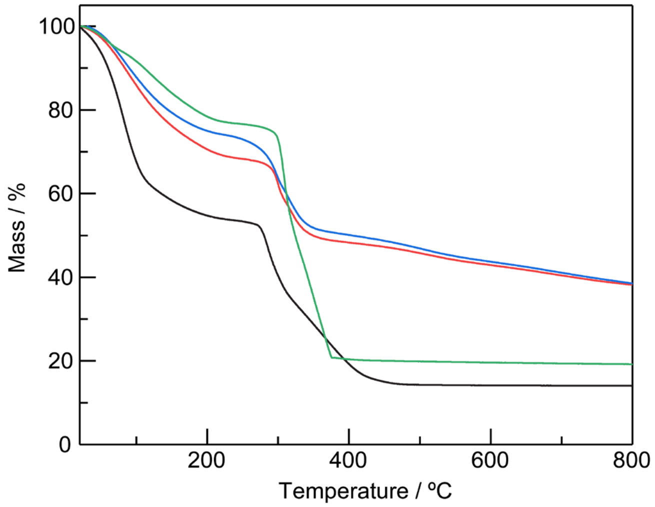

Figure S11. Thermogravimetric analysis plots of weight (%) versus temperature were recorded for the imine-linked **rht**-MOF obtained by tandem mechanochemical synthesis (—), mechanochemical synthesis using the preformed imine ligand (—), solvothermal synthesis modulated by HOAc (—), and solvothermal synthesis modulated by conc. HNO_3 (—). All samples exhibit comparable structural collapse temperatures of approximately 280 °C.

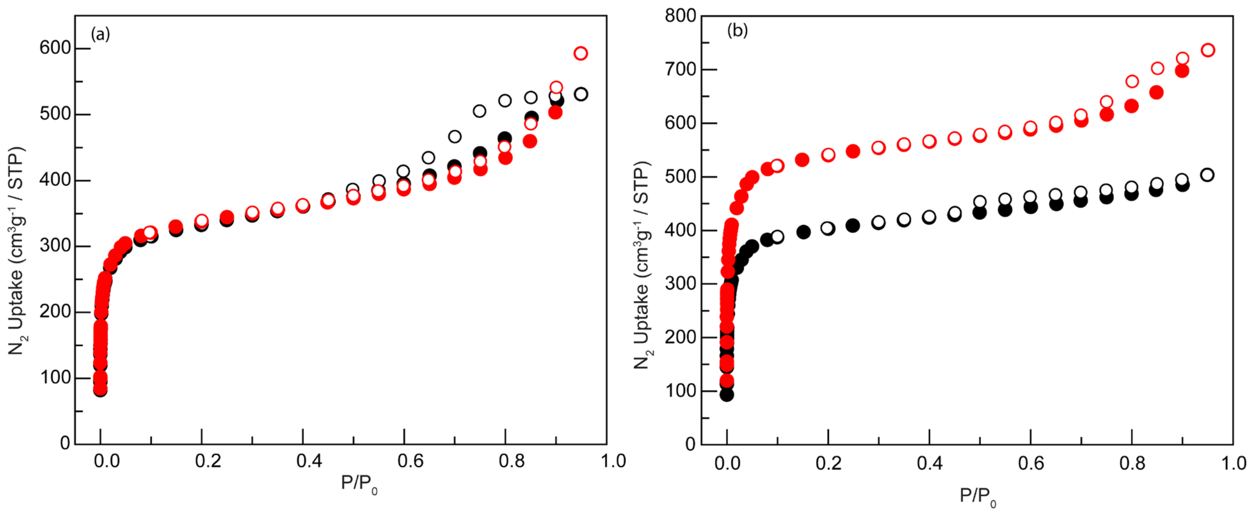

Figure S12. (a) N_2 adsorption isotherms at 77 K were collected the imine-linked **rht**-MOF obtained by solvothermal synthesis using the preform imine-based ligand, modulated by HOAc (adsorption (●), desorption (○)) or conc. HNO_3 (adsorption (●), desorption (○)). (b) N_2 adsorption isotherms at 77 K were also collected for the imine-linked **rht**-MOF obtained by mechanochemical synthesis using the preform imine-based ligand after 56 s (adsorption (●), desorption (○)) and 90 min (adsorption (●), desorption (○)).

Table S14. Surface area values of the solvothermally and mechanochemically obtained imine-linked **rht**-MOF using the preformed imine-based ligand were determined from N_2 adsorption isotherms measured at 77 K.

Entry	Conditions	BET surface area / m^2/g (P/P_0 = 0.007-0.03)	Langmuir surface area / m^2/g (P/P_0 = 0.007-0.03)
1	solvothermal synthesis with HOAc	1244	1296
2	solvothermal synthesis with conc. HNO_3	1265	1317
3	mechanochem. after 56 s	1523	1585
4	mechanochem. after 90 min	2051	2133

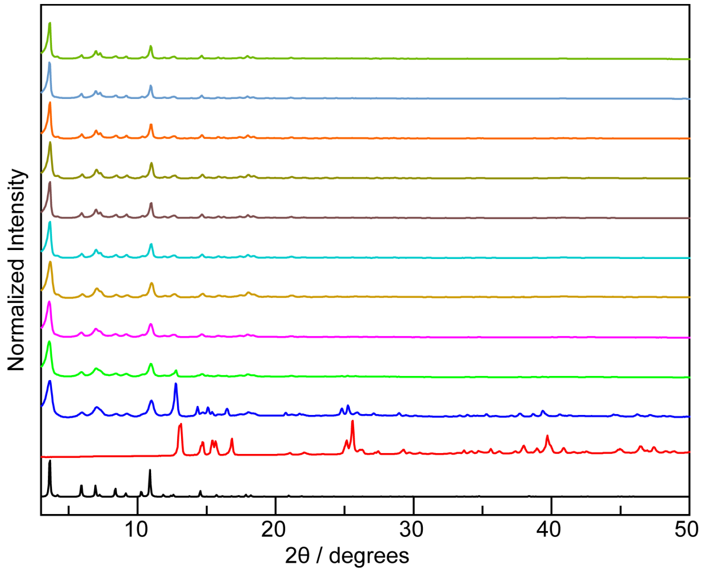

Figure S13. PXRD patterns were collected from experiments varying milling time for the tandem mechanochemical synthesis of imine-linked **rht**-MOF in the presence of DMF additive (η = 0.60 μL/mg, 58 μL): —, simulated; —, experimental $Cu(OAc)_2·H_2O$; —, *t* = 14 s; —, *t* = 28 s; —, *t* = 56 s; —, *t* =1.85 min; —, *t* = 3.75 min; —, *t* = 7.5 min; —, *t* = 15 min; —, *t* = 30 min; —, *t* = 60 min; —, *t* = 90 min. Milling time ≥ to 56s produced similar desired PXRD patterns.

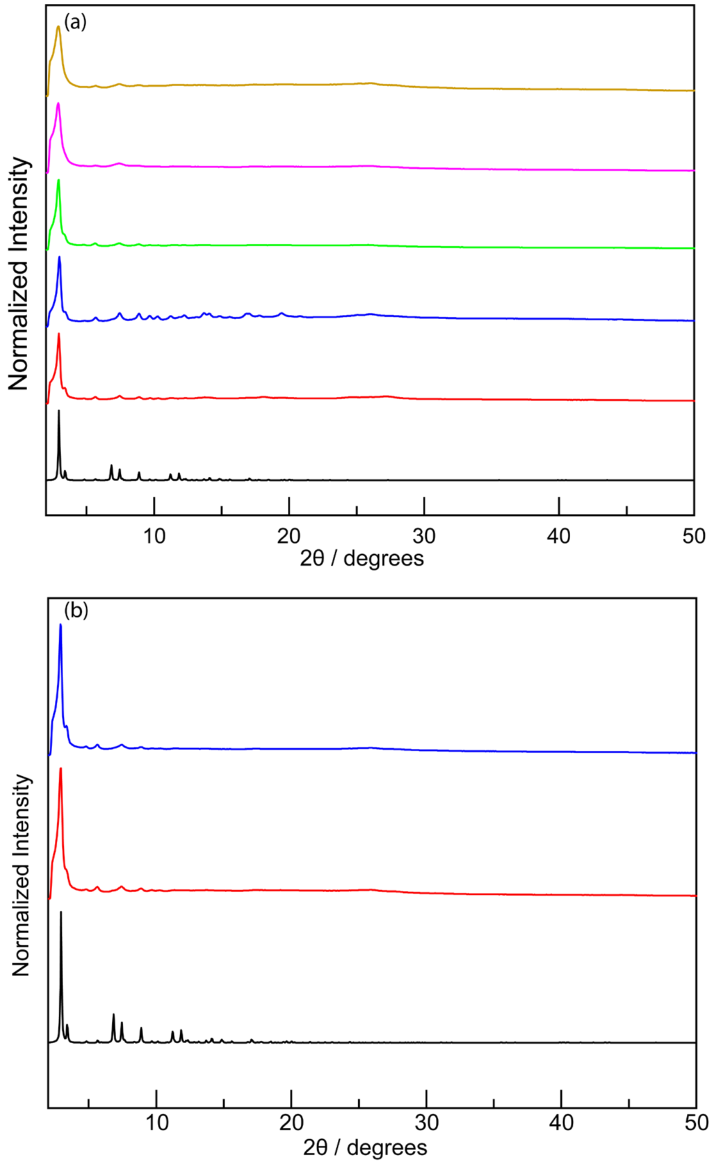

Figure S14. (a) PXRD patterns were collected from experiments varying the amount of DMF additive in the tandem mechanochemical synthesis of expanded imine-linked **rht**-MOF for 60 min: —, simulated; —, η = 0.30 μL/mg; —, η = 0.45 μL/mg; —, η = 0.60 μL/mg; —, η = 0.75 μL/mg; —, η = 0.91 μL/mg. Amounts of DMF ≤ 0.60 μL/mg produced similar well-defined PXRD patterns. (b) PXRD patterns were collected from experiments varying milling time in the tandem mechanochemical synthesis of expanded imine-linked **rht**-MOF in the presence of DMF additive (η = 0.60 μL/mg, 85 μL): —, simulated; —, t = 60 min; —, t = 90 min. Both milling times produced similarly well-defined PXRD patterns.

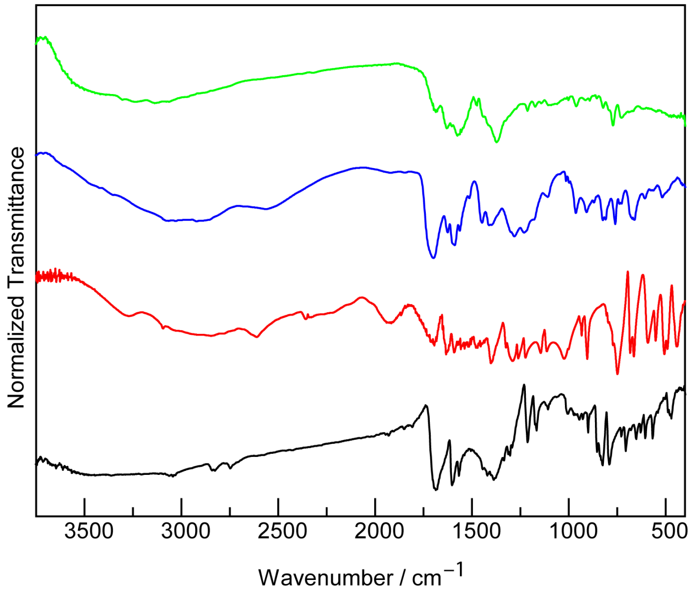

Figure S15. IR spectra were collected from 1,3,5-tris(4-formylphenyl)benzene (—), 5-aminoisophthalic acid (—), the pre-formed expanded imine-linked hexacarboxylic ligand (—), and the expanded imine-linked **rht**-MOF obtained by tandem mechanochemical synthesis (—). The coordinated C=O stretch observed in the MOF at 1372 cm^{-1} illustrates the formation of dative bonds.

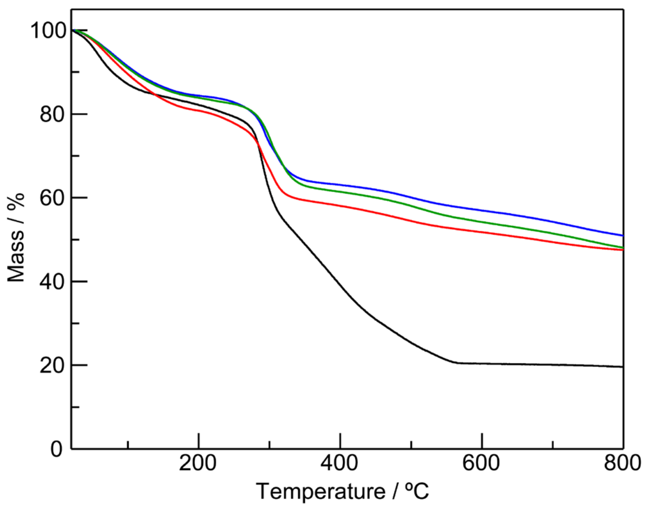

Figure S16. Thermogravimetric analysis plots of weight (%) versus temperature were recorded for the imine-linked expanded **rht**-MOF obtained by tandem mechanochemical synthesis (—), mechanochemical synthesis using the preformed imine ligand (—), solvothermal synthesis modulated by HOAc (—), and solvothermal synthesis modulated by conc. HNO_3 (—). All samples exhibit comparable structural collapse temperatures of approximately 270 °C.

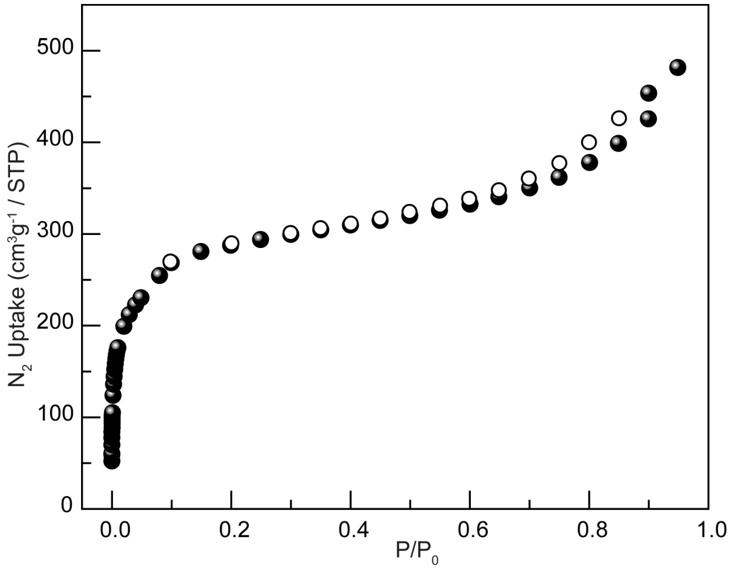

Figure S17. N_2 adsorption isotherm at 77 K was collected for the mechanochemically obtained expanded imine-linked **rht**-MOF (adsorption (●), desorption (○)).

Table S15. Surface area values of the mechanochemically obtained expanded imine-linked **rht**-MOF were determined from N_2 adsorption isotherm.

BET surface area / m^2/g (P/P_0 = 0.007-0.03)	Langmuir surface area / m^2/g (P/P_0 = 0.007-0.03)
976	1018

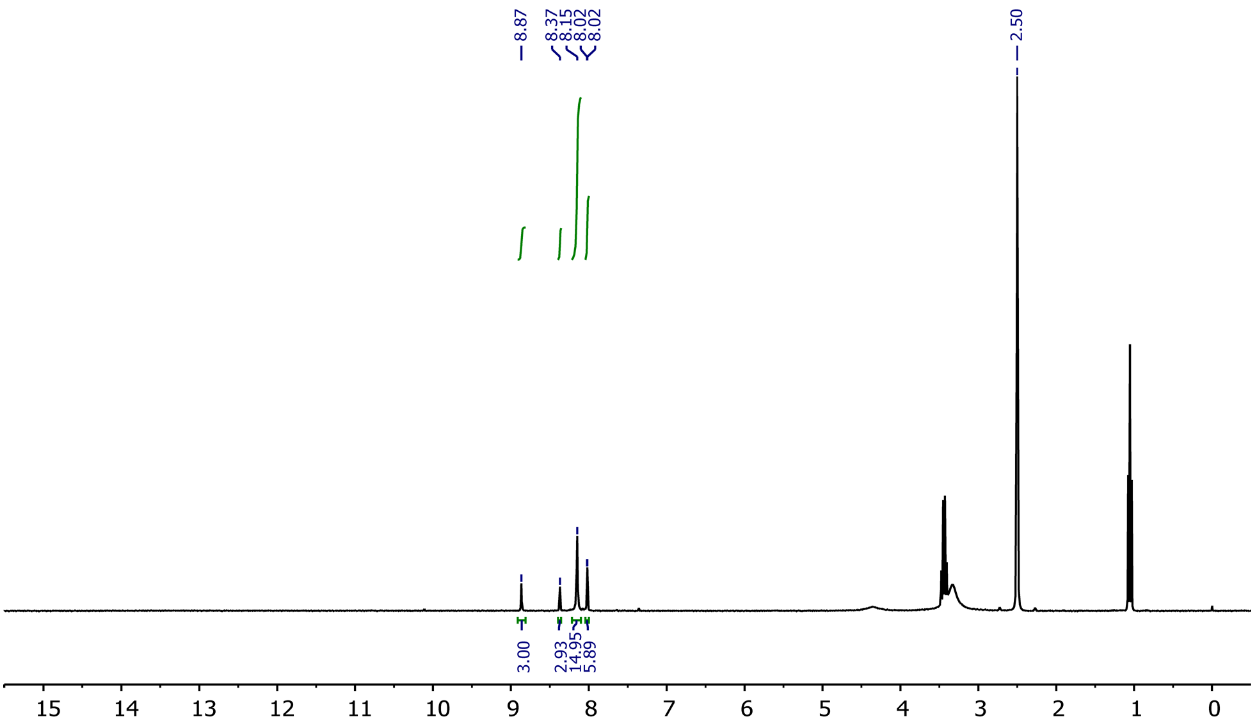

Figure S18. ^{1}H NMR spectrum of the imine-based *expanded* hexacarboxylic ligand obtained by the solution synthesis was acquired in d_6-DMSO at 23 °C.

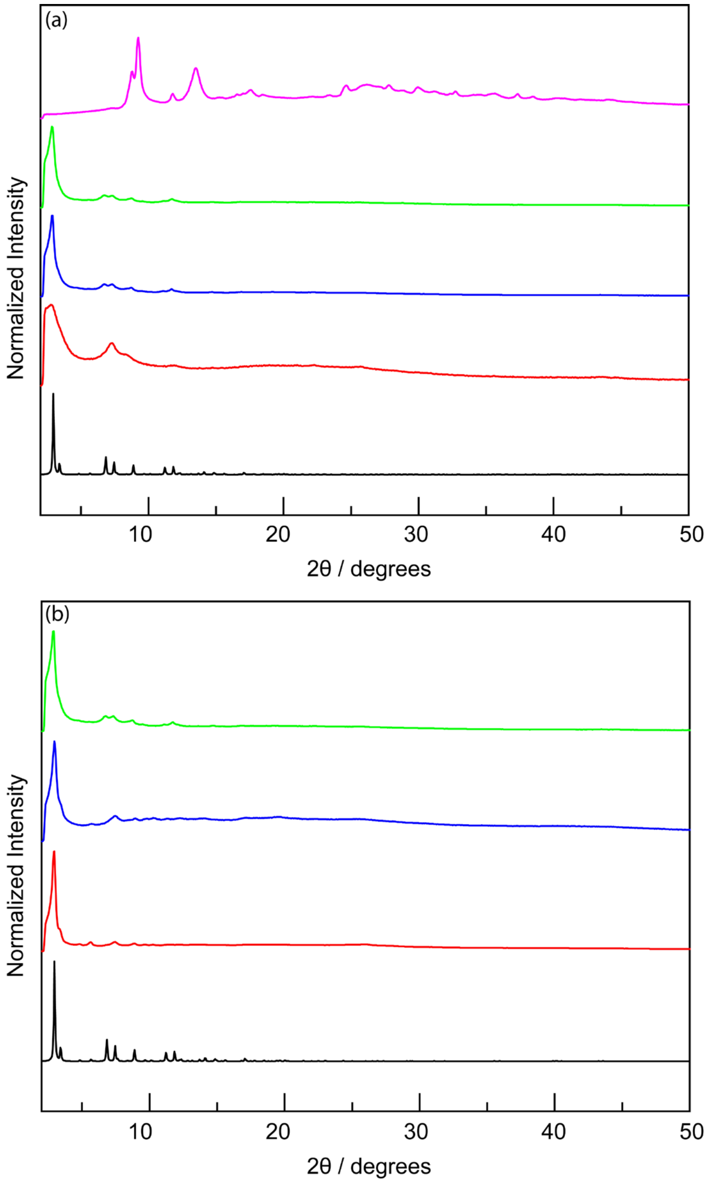

Figure S19. (a) PXRD patterns of the imine-linked expanded **rht**-MOF synthesized solvothermally from the pre-synthesized imine-based hexacarboxylic ligand, conducted at 65 °C for 72 h: —, simulated; —, without an acid modulator; —, HOAc; —, conc. HNO_3; —, 1 M aqueous HNO_3. (b) PXRD patterns of the imine-linked expanded **rht**-MOF obtained by tandem mechanochemical synthesis (—) and by mechanochemical synthesis using the pre-synthesized imine ligand (—), compared with the simulated pattern (—) and the solvothermal product modulated by HOAc (—).

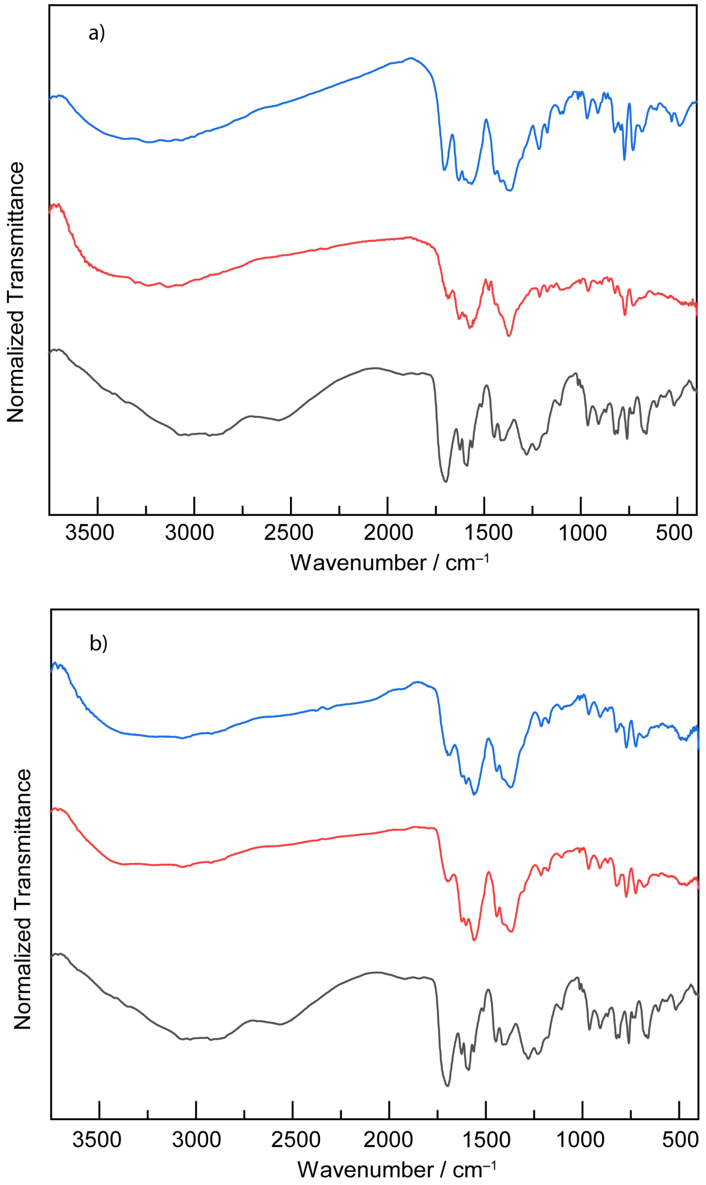

Figure S20. (a) IR spectra were collected from the preformed imine-based *expanded* hexacarboxylic ligand (—) and the imine-linked *expanded* **rht**-MOF obtained by tandem mechanochemical synthesis (—) and by mechanochemical synthesis using the preform ligand (—). (b) IR spectra were collected from the imine-linked *expanded* **rht**-MOF obtained by solvothermal synthesis modulated with HOAc (—) and conc. HNO_3 (—), compared to the preformed imine ligand (—).

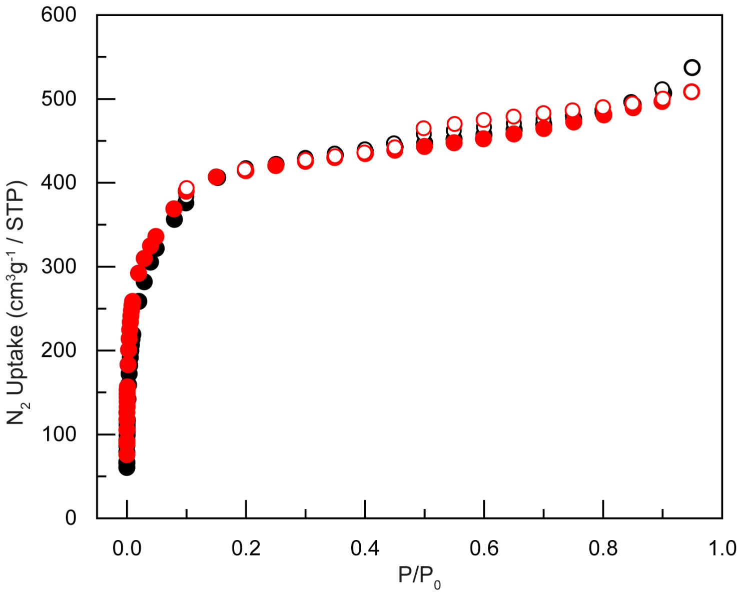

Figure S21. N_2 adsorption isotherms at 77 K were collected the imine-linked **rht**-MOF obtained by solvothermal synthesis using the preform imine-based ligand, modulated by HOAc (adsorption (●), desorption (○)) and by mechanochemical synthesis using the preform imine-based ligand (adsorption (●), desorption (○)).

Table S16. Surface area values of the solvothermally and mechanochemically obtained imine-linked **rht**-MOF using the preformed imine-based ligand were determined from N_2 adsorption isotherms measured at 77 K.

Entry	Synthesis conditions	BET surface area / m^2/g (P/P_0 = 0.007-0.03)	Langmuir surface area / m^2/g (P/P_0 = 0.007-0.03)
1	solvothermal synthesis with HOAc	1349	1411
2	mechanochemical synthesis	1421	1482

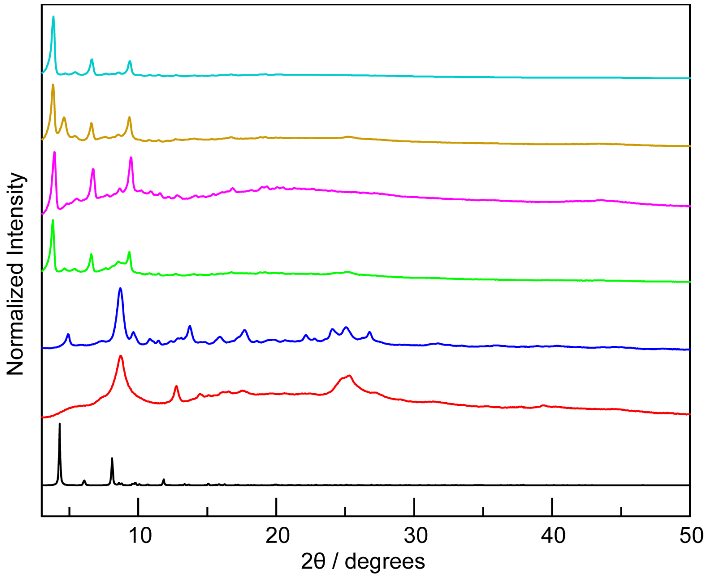

Figure S22. PXRD patterns were collected from experiments varying the amount of DMF additive in the mechanochemical synthesis of imine-linked **pto**-MOF using 90-min milling at 30 Hz: —, simulated; —, η = 0.15 µL/mg; —, η = 0.30 µL/mg; —, η = 0.45 µL/mg; —, η = 0.60 µL/mg; —, η = 0.75 µL/mg; —, η = 0.90 µL/mg. The sample with η = 0.90 µL/mg DMF produced the best PXRD pattern.

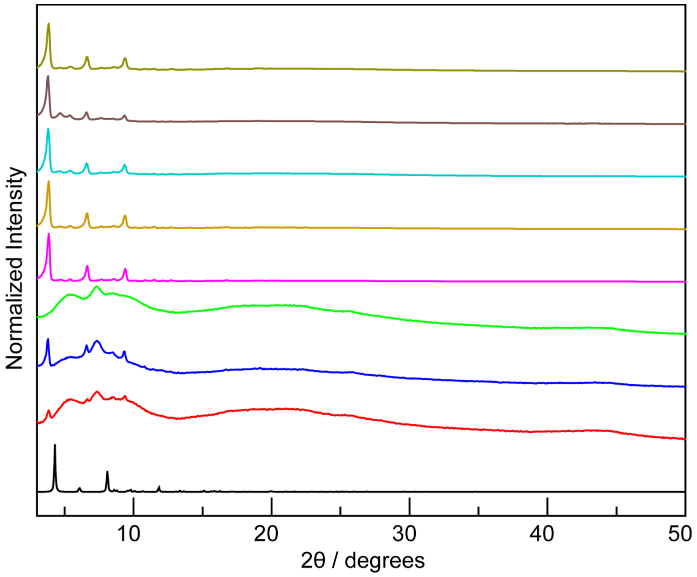

Figure S23. PXRD patterns were collected from experiments varying milling time in the mechanochemical synthesis of imine-linked **pto**-MOF in the presence of DMF additive (η = 0.90 μL/mg, 120 μL) at 30 Hz: —, simulated; —, t = 7.5 min; —, t = 15 min; —, t = 23 min; —, t = 30 min; —, t =45 min; —, t = 60 min; —, t = 75 min; —, t = 90 min. Milling time ≥ 30min produced similarly well-defined PXRD patterns.

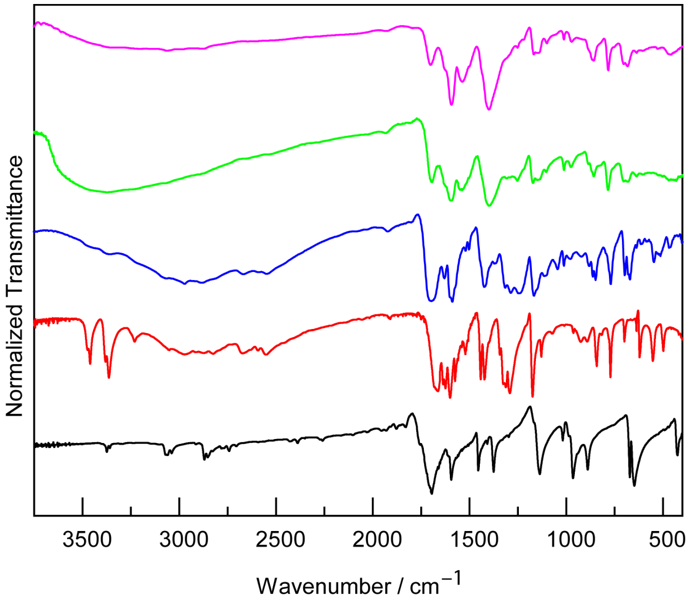

Figure S24. IR spectra were collected from benzene-1,3,5-tricarboxaldehyde (—), 4-aminobenzoic acid (—), the imine-based tricarboxylic ligand (—), and the imine-linked **pto**-MOF obtained by tandem mechanochemical synthesis (—) and by mechanochemical synthesis using the preformed imine ligand (—). The coordinated C=O stretch observed at 1400 cm^{-1} in the resultant MOFs illustrates the formation of dative bonds. The disappearance of N–H stretches between 3473 cm^{-1} and 3364 cm^{-1} from the primary amine group highlights its conversion to imine bonds.

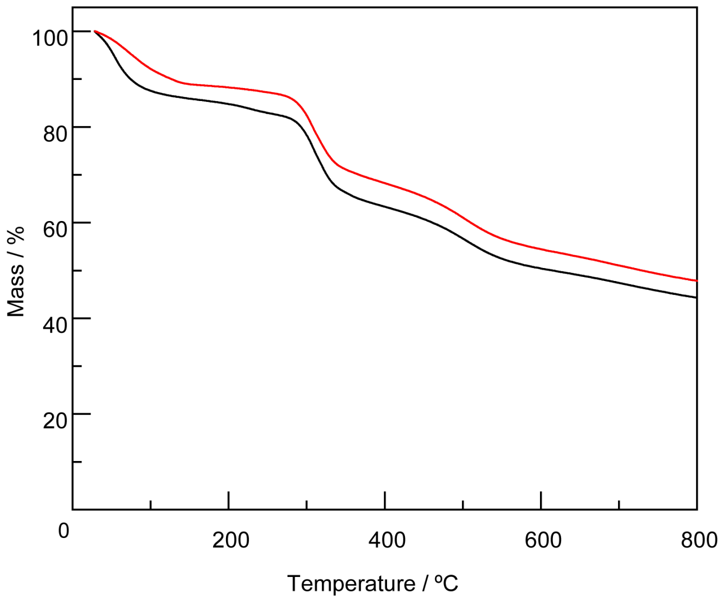

Figure S25. Thermogravimetric analysis plots of weight (%) versus temperature were recorded for the imine-linked **pto**-MOF obtained by tandem mechanochemical synthesis (—) and by mechanochemical synthesis using the preformed imine ligand (—).

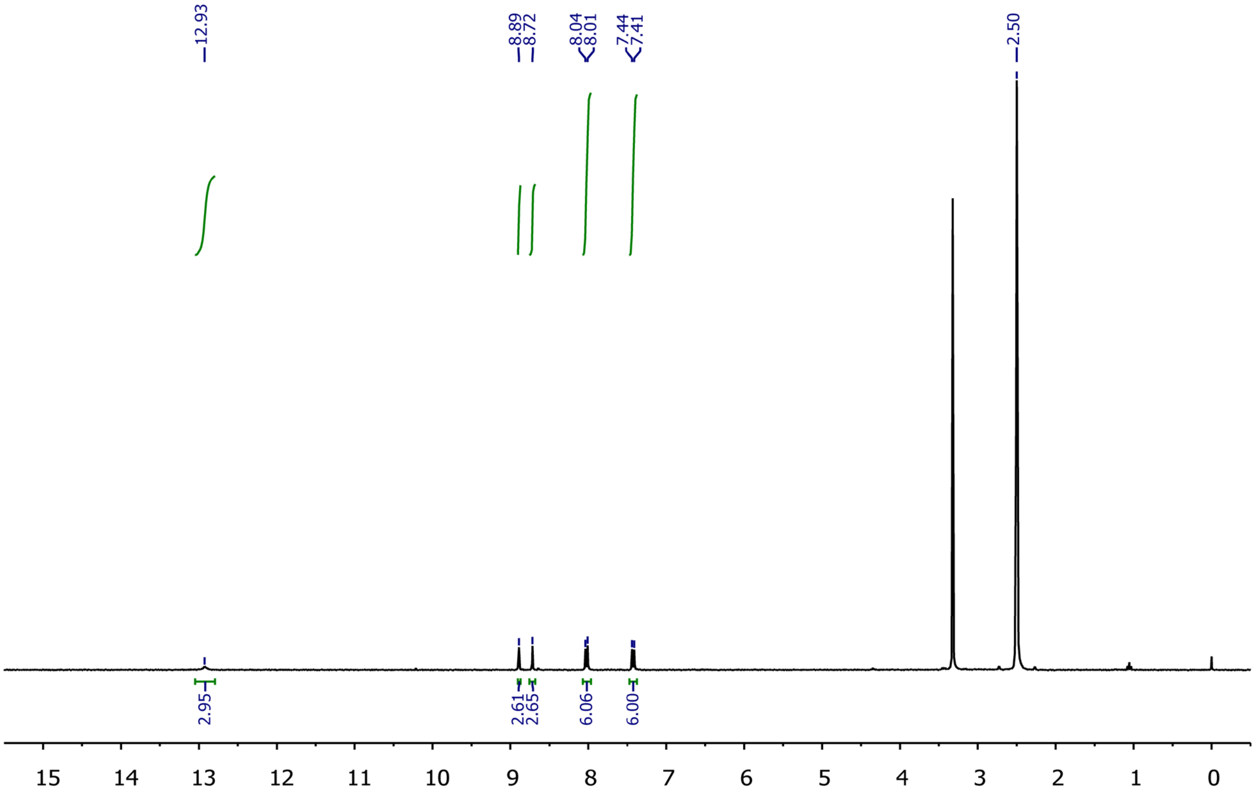

Figure S26. ^{1}H NMR spectrum of the imine-based tricarboxylic ligand (4,4',4''-((benzene-1,3,5-triyltris(methaneylylidene))tris(azaneylylidene))tribenzoic acid) obtained by the solution synthesis was acquired in d_6-DMSO at 23 °C.

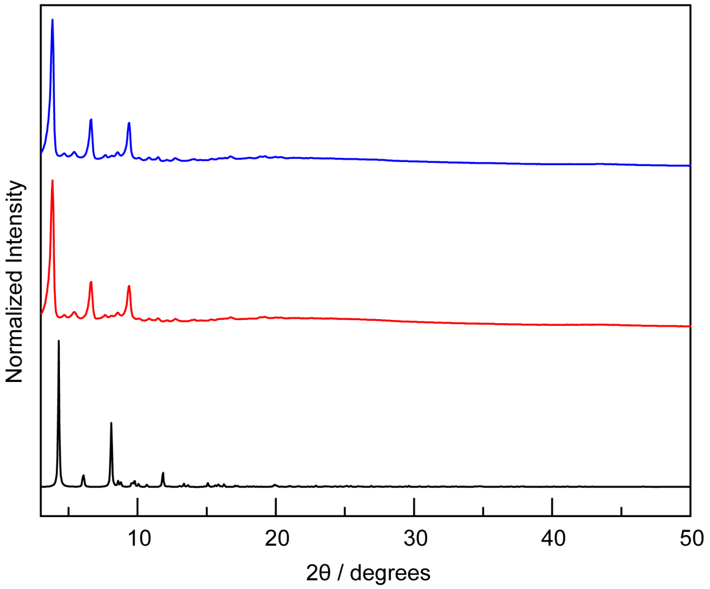

Figure S27. PXRD patterns of the imine-linked **pto**-MOF obtained by tandem mechanochemical synthesis (—) and by mechanochemical synthesis using the preformed imine ligand (—), compared with the simulated pattern (—).

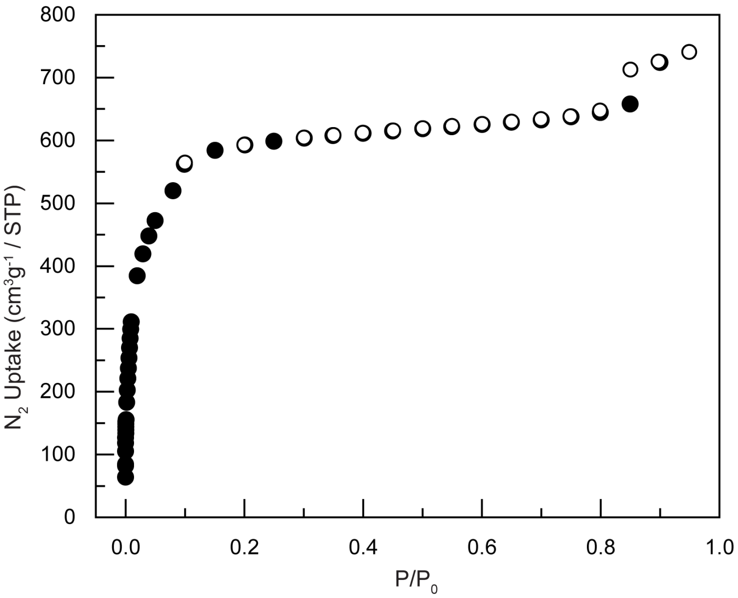

Figure S28. N_2 adsorption isotherm at 77 K was collected the imine-linked **pto**-MOF obtained by mechanochemical synthesis using the preform imine-based ligand (adsorption (●), desorption (○)).

Table S17. Surface area values of the mechanochemically obtained imine-linked **pto**-MOF using the preformed imine-based ligand were determined from N_2 adsorption isotherms measured at 77 K.

Entry	Synthesis conditions	BET surface area / m²/g (P/P_0 = 0.007-0.03)	Langmuir surface area / m²/g (P/P_0 = 0.007-0.03)
1	mechanochemical synthesis	2105	2210

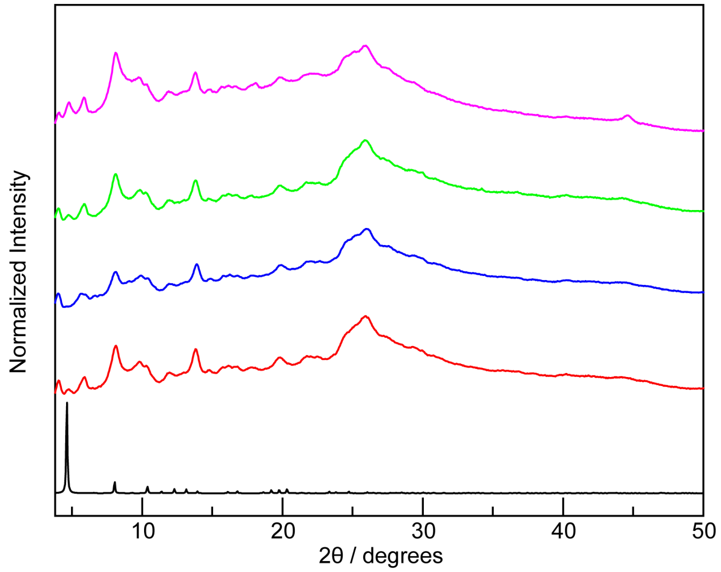

Figure S29. PXRD patterns were collected from experiments examining the amount of DMF additive and milling time at 30 Hz in attempts to synthesize the amide-linked **pto**-MOF mechanochemically, without success: —, simulated; —, η = 0.60 μL/mg, t = 60 min; —, η = 0.90 μL/mg, t = 60 min; —, η = 0.60 μL/mg, t = 90 min, —, η = 0.90 μL/mg, t = 90 min.

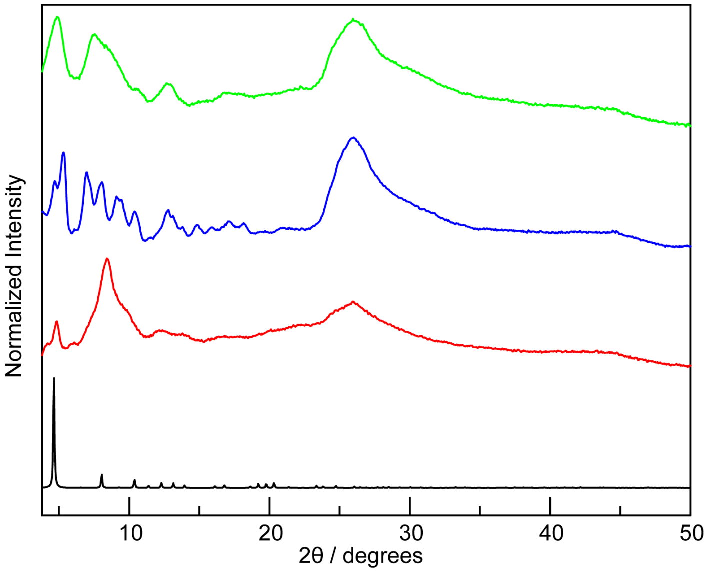

Figure S30. PXRD patterns were collected from experiments testing different liquid additives (DMF, MeOH, and H_2O) in attempts to synthesize the amide-linked **pto**-MOF mechanochemically, without success (η = 0.60 μL/mg, 48 μL, *t* = 90 min): —, simulated; —, η = DMF; —, η = MeOH; —, η = H_2O.

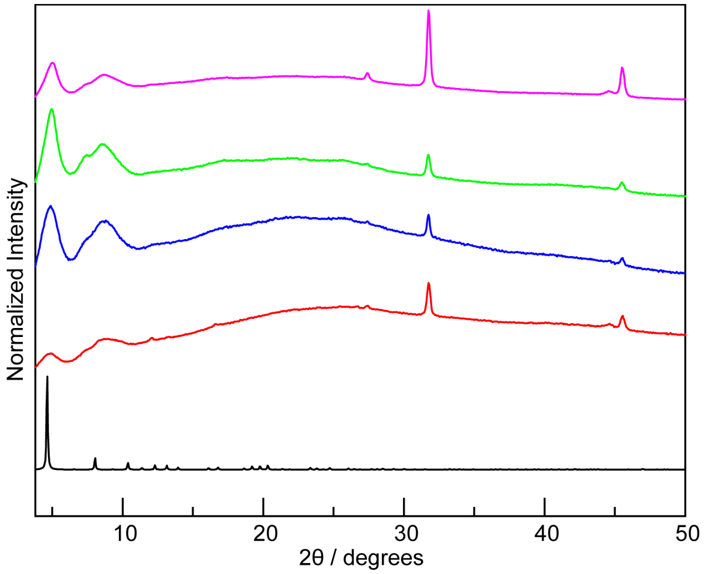

Figure S31. PXRD patterns were collected from experiments varying the amount of DMF additive in attempts to synthesize the alkyne-linked **pto**-MOF mechanochemically using 90-min milling at 30 Hz, without success: —, simulated; —, η = 0.31 μL/mg; —, η = 0.60 μL/mg; —, η = 0.75 μL/mg; —, η = 0.90 μL/mg.

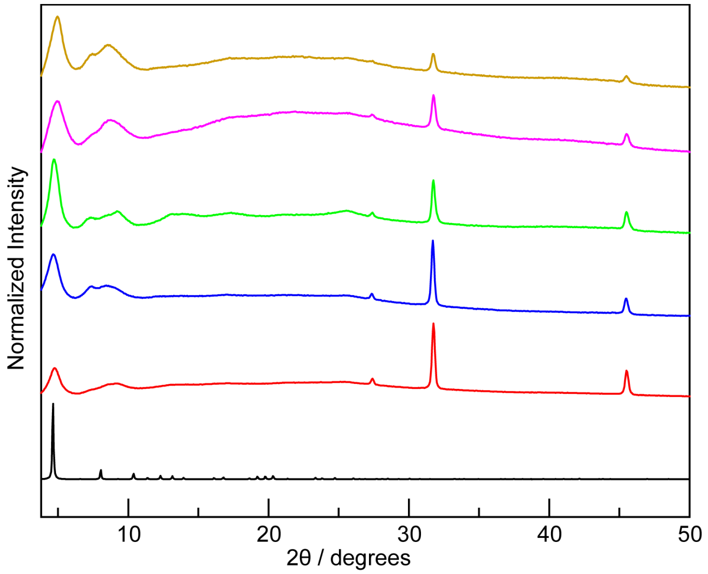

Figure S32. PXRD patterns were collected from experiments varying milling time in attempts to synthesize the alkyne-linked **pto**-MOF mechanochemically in the presence of DMF additive (η = 0.75 μL/mg, 56 μL) at 30 Hz, without success: —, simulated; —, *t* = 7.5 min; —, *t* = 15 min; —, *t* = 30 min; —, *t* = 60 min; —, *t* = 90 min.

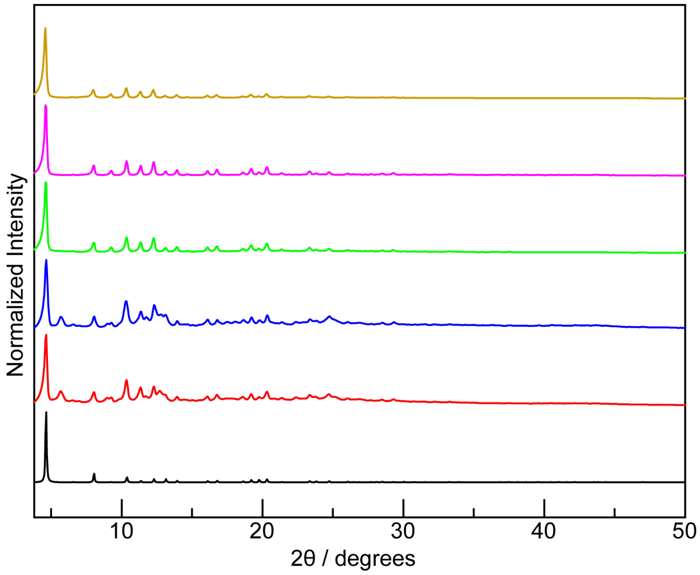

Figure S33. PXRD patterns were collected from experiments varying the amount of EtOH additive in the mechanochemical synthesis of MOF-14 using 45-min milling at 30 Hz: —, simulated; —, η = 0.30 μL/mg; —, η = 0.45 μL/mg; —, η = 0.60 μL/mg, —, η = 0.90 μL/mg; —, η = 1.2 μL/mg. Amounts of EtOH ≥ 0.60 μL/mg produced similarly well-defined PXRD pattern.

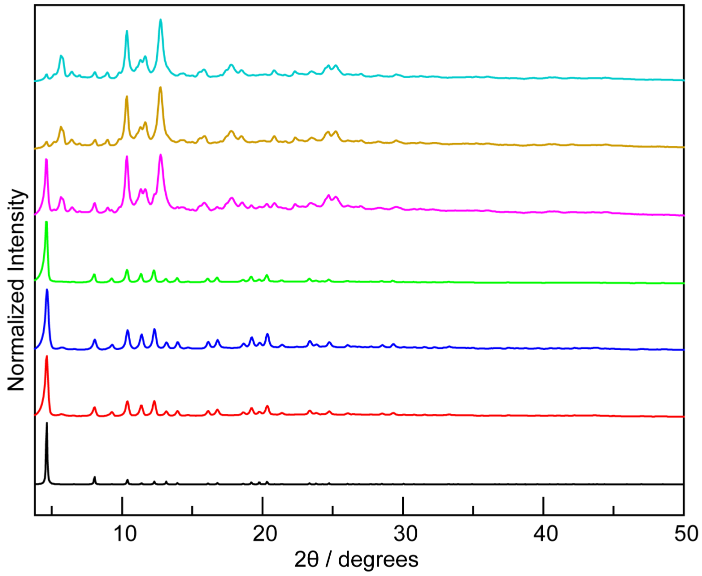

Figure S34. PXRD patterns were collected from experiments varying milling time in the mechanochemical synthesis of MOF-14 in the presence of EtOH additive (η = 0.60 μL/mg, 77 μL) at 30 Hz: —, simulated; —, *t* = 15 min; —, *t* = 30 min; —, *t* = 45 min, —, *t* = 60 min; —, *t* = 75 min; —, *t* = 90 min. Milling times between 15 and 45 min produced similarly well-defined PXRD patterns.

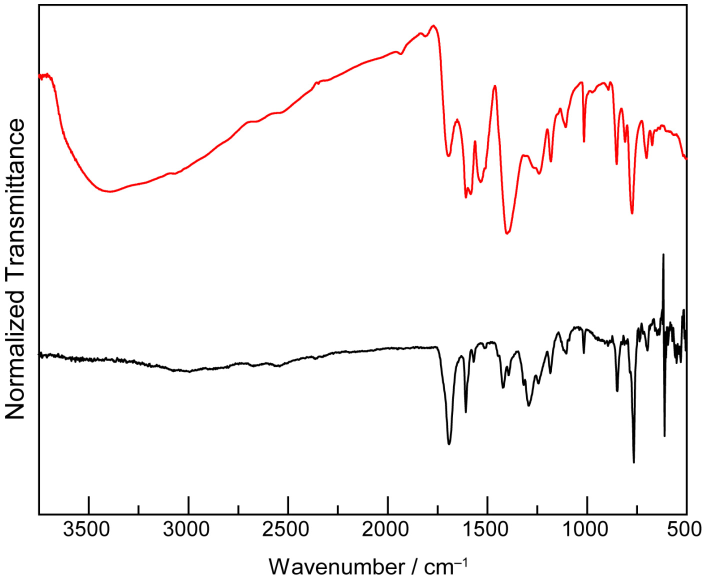

Figure S35. IR spectra (3750–500 cm^{-1}) were collected from 1,3,5-tris(4-carboxyphenyl) benzene (—) and the mechanochemically obtained MOF-14 (—). The coordinated C=O stretch observed at 1397 cm^{-1} in the resultant MOF illustrates the formation of dative bonds.

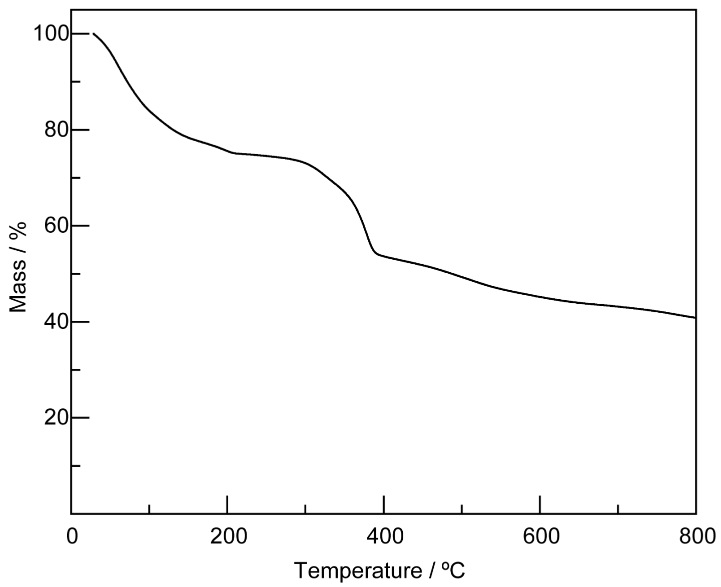

Figure S36. A plot of weight% vs. temperature was recorded by thermogravimetric analysis of the mechanochemically obtained MOF-14 (—).

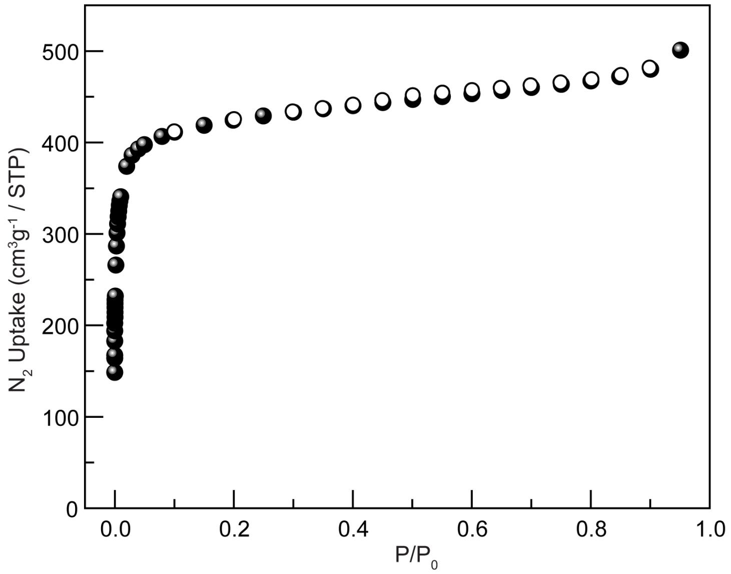

Figure S37. N_2 adsorption isotherm at 77 K was collected from the mechanochemically obtained MOF-14 (adsorption (●), desorption (○)).

Table S18. Surface area values of the mechanochemically obtained MOF-14 were determined from N_2 adsorption isotherm.

BET surface area / m^2/g (P/P_0 = 0.007-0.03)	Langmuir surface area / m^2/g (P/P_0 = 0.007-0.03)
1719	1790

D. References

1. Wang, C.-H.; Gao, W.-Y.; Ma, Q.; Powers, D. C., Templating metastable Pd_2 carboxylate aggregates. *Chem. Sci.*, **2019**, *10*, 1823-1830.
2. Aharoni, S. M.; Edwards, S. F., Gels of rigid polyamide networks. *Macromolecules*, **1989**, *22*, 3361-3374.
3. Fulmer, G. R.; Miller, A. J. M.; Sherden, N. H.; Gottlieb, H. E.; Nudelman, A.; Stoltz, B. M.; Bercaw, J. E.; Goldberg, K. I., NMR Chemical Shifts of Trace Impurities: Common Laboratory Solvents, Organics, and Gases in Deuterated Solvents Relevant to the Organometallic Chemist. *Organometallics* **2010**, *29*, 2176-2179.
4. Macrae, C. F.; Edgington, P. R.; McCabe, P.; Pidcock, E.; Shields, G. P.; Taylor, R.; Towler, M.; van de Streek, J., Mercury: visualization and analysis of crystal structures. *J. Appl. Crystallogr.*, **2006**, *39*, 453-457.
5. Mardiansyah, D.; Badloe, T.; Triyana, K.; Mehmood, M. Q.; Raeis-Hosseini, N.; Lee, Y.; Sabarman, H.; Kim, K.; Rho, J., Effect of temperature on the oxidation of Cu nanowires and development of an easy to produce, oxidation-resistant transparent conducting electrode using a PEDOT:PSS coating. *Sci. Rep.* **2018**, *8*, 10639.
6. Klimakow, M.; Klobes, P.; Thünemann, A. F.; Rademann, K.; Emmerling, F., Mechanochemical Synthesis of Metal–Organic Frameworks: A Fast and Facile Approach toward Quantitative Yields and High Specific Surface Areas. *Chem. Mater.*, **2010**, *22*, 5216-5221.
7. Klimakow, M.; Klobes, P.; Rademann, K.; Emmerling, F., Characterization of mechanochemically synthesized MOFs. *Microporous Mesoporous Mater.*, **2012**, *154*, 113-118.
